# Surfaceome: a new era in the discovery of immune evasion mechanisms of circulating tumor cells

**DOI:** 10.1002/1878-0261.13665

**Published:** 2024-05-22

**Authors:** Doryan Masmoudi, Jérome Vialaret, Christophe Hirtz, Catherine Alix‐Panabières

**Affiliations:** ^1^ Laboratory of Rare Circulating Human Cells University Medical Center of Montpellier France; ^2^ University of Montpellier, IRMB‐PPC, INM, CHU Montpellier, INSERM CNRS France; ^3^ CREEC/CANECEV, MIVEGEC (CREES), University of Montpellier, CNRS, IRD France; ^4^ European Liquid Biopsy Society (ELBS) Hamburg Germany

**Keywords:** cancer, circulating tumor cells, glycoproteomics, immunopeptidomics, proteomics

## Abstract

Circulating tumor cells (CTCs) are cancer cells that detach from the original site and reach the bloodstream. The most aggressive CTCs survive various immune system attacks and initiate metastasis formation. Importantly, CTCs are not specifically targeted by the current immunotherapies due to the limited knowledge on specific targets. Proteomic profiling can be a powerful tool for understanding some of the immune evasion mechanisms used by cancer cells and particularly CTCs. These mechanisms are generally linked to the expression of specific surface proteins/peptides (i.e. the surfaceome). The study of the peptides that bind to class I molecules of the major histocompatibility complex (MHC‐I) and of the various glycoproteins expressed on CTC surface may open a completely new avenue for the discovery of novel mechanisms of immune evasion. In this review, we discuss how immunopeptidomic and glycoproteomic studies of CTCs that interact with immune cells could help to better understand how metastasis‐initiator CTCs escape the host immune response. We also describe how immunopeptidomic and glycoproteomic studies are carried out.

## Graphical abstract

In the era of immunotherapies, many patients either do not respond or eventually develop resistance. We propose to pave the way for proteomic analysis of surface‐expressed proteins called surfaceome, of circulating tumor cells. This approach seeks to identify immune evasion mechanisms and discover potential therapeutic targets.

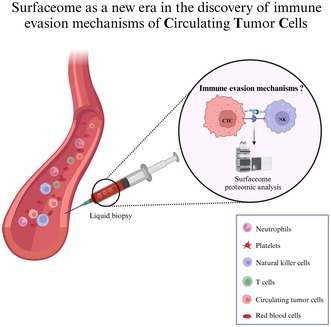

**Abbreviations**
CAFs: cancer‐associated fibroblasts
CAR‐T: chimeric antigen receptor T cells
CK: cytokeratin
CTCs: circulating tumor cells
CTLs: cytotoxic T lymphocytes
DCs: dendritic cells
DDA: data‐dependent acquisition
DIA: data‐independent acquisition
ECM: extracellular matrix
EMA: European Medicines Agency
EMT: epithelial‐to‐mesenchymal transition
EVs: extracellular vesicles
FASL: Fas ligand
FDA: Food and Drugs Administration
FGF: fibroblast growth factor
HLA: human leukocyte antigen
ICIs: immune checkpoint inhibitors
LC: liquid chromatography
MDSCs: myeloid‐derived suppressor cells
MHC‐I: major histocompatibility complex class I
MMP: matrix metalloproteinase
MS: mass spectrometry
NETs: neutrophil extracellular traps
NK: natural killer
PADI: protein arginine deiminase
PD‐1: programmed cell death protein 1
PD‐L1: programmed death‐ligand 1
PMN‐MDSCs: polymorphonuclear‐MDSCs
PTMs: post‐translational modifications
SIGLEC: sialic acid‐binding immunoglobulin‐type lectin
TAA: tumor‐associated antigen
TAP: transporter associated with antigen processing
TCR: T cell receptor
TGF: transforming growth factor
Treg: regulatory T cells
TSA: tumor‐specific antigen
VEGF: vascular endothelial growth factor

## 1 Introduction

Cancer metastasis is the leading cause of death and remains a serious challenge despite the many efforts to improve its diagnosis, therapy and follow‐up [1, 2]. The metastatic process is a cascade of events that include the invasion, intravasation, and dissemination of few cancer cells in the bloodstream and their subsequent colonization of distant organs [3]. Once cancer cells enter the bloodstream, they are called circulating tumor cells (CTCs) [4]. During the process of intravasation, CTCs leave their protective tumor microenvironment, become vulnerable to the host immune cells and are exposed to different environmental insults (e.g. mechanical stress and the pressure exerted by the blood flow) [5]. Therefore, most cancer cells entering the bloodstream do not survive [6]. On the other hand, CTCs that have overcome the host immune system attacks and shear stress constitute a more aggressive subgroup of cells that could reach many distant organs. Liquid biopsy has paved the way to understand CTC biological properties and has led to major discoveries in cancer [7]. This term was coined by Catherine Alix‐Panabières and Klaus Pantel in 2010 to describe a blood test used for CTC‐based semi‐invasive cancer monitoring [8]. Then, liquid biopsy has been extended to other analytes, such as circulating tumor DNA [9], extracellular vesicles (EVs) [10], tumor‐educated platelets [11], circulating cell‐free RNA [12], and circulating proteins [13]. This assay is an ideal companion tool in the framework of personalized medicine that will be implemented in the next decades [14].

Many efforts have been undertaken to detect CTCs by liquid biopsy in order to use them as prognostic biomarkers and to guide therapy decision‐making [13]. As a result, CTCs from different cancer types have been phenotypically characterized to acquire a better knowledge of their function and survival potential. Specifically, it has been established that their phenotype can be different from that of the original tumor and that this confers to CTCs immune escape capabilities [15]. Moreover, due to such phenotypic differences, CTCs may not respond to personalized therapies that target molecules expressed on the primary tumor cell surface. Therefore, studies on CTC immune evasion mechanisms are required. CTCs are frequently characterized with genomic and transcriptomic techniques, but very rarely using proteomic tools. Yet, proteomic profiling of CTCs could bring major insights into the mechanisms of self‐recognition by the immune system and into the ligands implicated in immune evasion. This might lead to the identification of new targets for personalized therapies. Immunopeptidomics is gaining interest in the age of mRNA‐ and peptide‐based vaccines as well as chimeric antigen receptor T cells (CAR‐T). This entails the investigation of the immunomodulatory peptides (including tumor‐specific antigen‐derived peptides or neo‐peptides) that bind to class 1 molecules of the major histocompatibility complex class 1 (MHC‐I) [16]. In the context of cancer, neo‐peptides are important for self‐immunosurveillance by CD8^+^ T cells and are central to the quest of new targetable cancer‐specific antigens [17]. Recent advances in liquid chromatography (LC) and mass spectrometry (MS) have allowed identifying some MHC‐bound peptides that induce immunological responses [18] and better understanding of how immunological surveillance (i.e. recognition of self *vs* non‐self) works. However, CTC recognition as self by T cells does not fully explain CTC immune evasion mechanisms.

Glycoproteins also are essential interfaces with the immune system and their glycosylation levels and sites regulate these interactions [19]. Aberrant glycosylation is currently considered one of the hallmarks of cancer because it influences cancer aggressiveness and capacity to avoid immunosurveillance [20]. As glycoproteins interact with a wide range of immune cells, such as natural killer (NK) and T cells, they have immunomodulatory properties [21]. Although analyzing glycosylation sites and levels is challenging, glycoproteomics is rapidly developing [22].

In this review, we will describe the role of CTCs in the metastatic cascade and discuss the multiple routes they may take to evade the immune system. In order to identify these mechanisms, we propose the investigation of surface‐expressed proteins, the so‐called “surfaceome”, and suggest some proteomic‐based strategies to analyze them.

The main goal is to identify new therapeutic targets for developing new immunotherapies.

## 2 Circulating tumor cells and the metastatic process

CTCs are cancer cells of epithelial origin found in the blood of patients with cancer. They were first described by Thomas Ashworth who analyzed a blood sample from a patient with metastatic cancer under a microscope in 1869 [23]. He reached the conclusion that CTCs had made their way through the bloodstream from the primary cancer to the vein from which he collected the blood sample. During the metastatic process, these rare cells are released from the initial tumor site. The term metastatic cascade describes a multi‐step process: local invasion, intravasation, blood survival, extravasation, and colonization [24, 25]. The kinetics of this cascade are influenced by the organotropism of certain cells, the speed of progression of each cancer subtypes, and the nature of the interactions between cancer cells and the host stroma [26, 27].

Following cell‐to‐cell adhesion loss and extracellular matrix (ECM) breakdown, cancer cells start to undergo epithelial‐to‐mesenchymal transition (EMT) and develop a transient and partial mesenchymal phenotype that allows them to acquire resistance to apoptotic signals [25]. Such cells can initiate intravasation. This stage entails interactions with the endothelial cells that form the blood vessels. These interactions are orchestrated by EMT regulators. For example, ZEB1 (an E‐cadherin repressor) silencing in prostate cancer cells results in the decrease of N‐cadherin expression (normally upregulated in EMT) and the reduction of transendothelial migration [28]. Furthermore, when overexpressed, the transcription factor TWIST1 (an EMT inducer) can influence vascular mimicry whereby aggressive tumor cells can mimic the embryonic vasculogenic network by interacting with endothelial cells [29].

When they reach the bloodstream, cancer cells are exposed to a strong difference in pressure and few of them will survive. Indeed, although aggressive tumors can release thousands of cancer cells into the bloodstream, only few of them will colonize to a new site [30]. The few studies that tried to estimate the half‐life of breast CTCs in blood reported values ranging from 30 min to 2.4 h [31, 32]. It has been predicted that just one CTC in 10 000 can initiate metastasis [33]. However, CTCs may aggregate and form clusters to increase their chances of survival. Homotypic clusters are composed only of CTCs. Conversely, in heterotypic clusters, CTCs interact with different blood cell types, such as neutrophils and platelets [32, 34]. By traveling in the bloodstream in a cluster, CTC resistance to the immune system attacks is enhanced via paracrine signaling or direct contacts [35], as well as their protection against anoikis [36]. Therefore, CTC clusters have a stronger invasive potential than CTCs on their own [37]. However, CTC clusters have been mainly observed in blood samples from patients with metastatic cancer. Only one report mentioned their detection in patients with early‐stage breast cancer [38]. Lastly, during their dissemination, single (up to 20 μm in diameter) and clustered CTCs are stopped due to the reduction in the blood vessel size (3–7 μm), resulting very rarely in extravasation, a crucial stage in the metastatic cascade [39].

An important feature of the metastatic process is that cancer cells from specific tumor types appear to preferentially invade specific organs. For instance, CTCs from breast cancer have a strong affinity for lung, liver, bone, and brain. This organ selectivity is the logical consequence of the “seed and soil” theory introduced by Stephen Paget in 1889 [40]. Paget hypothesized that like a seed that will only develop in fertile soil, a metastatic cancer cell or cancer stem cell will only extravasate if the microenvironment is suitable. Recently, Welch and Hurst proposed a third concept, called “climate”, that refers to how each individual's overall health and genetic inheritance might influence metastasis formation [41].

To reach a new site, cancer cells must first adhere to a new barrier of endothelial cells in a process called extravasation. Selectins are a family of receptors that govern cancer cell interaction with endothelial cells. E‐selectin, a cytokine‐inducible endothelial cell adhesion receptor, has been implicated as a mediator of carcinoma cell interaction with endothelial cells and of metastasis to the liver [42]. Adherence to endothelial cells is influenced by the cancer cell ability to bind to selectins. This is governed by the glycosylation level of proteins at the cancer cell surface. Indeed, abnormal protein glycosylation is one of the hallmarks of cancer. Sialyl Lewis a and Sialyl Lewis x are carbohydrates that play a role in cancer cell adherence to vascular endothelium through selectins [43, 44]. Thus, accumulation of carbohydrate ligands on the cancer cell surface is strongly linked to the occurrence of hematogenous metastases. Selectin expression on the surface of endothelial cells is influenced not only by cancer cells, but also by specific cellular components and the secretion of specific compounds. For instance, myeloid‐derived suppressor cells (MDSC; a type of immune cells) can increase E‐selectin expression on the surface of endothelial cells through interleukin (IL)‐1 secretion, contributing to the establishment of the pre‐metastatic niche [45]. Moreover, activated platelets can secrete chemotactic factors, such as CXCL5/7, leading to the recruitment of granulocytes, an essential component of the future metastatic niche [46].

Fibroblasts also play a role in remodeling the ECM and in establishing the pre‐metastatic niche. Their differentiation into cancer‐associated fibroblasts (CAFs) and the release of matrix metalloproteinases (MMPs) allow them to prepare the environment for cancer cell invasion [47]. Tumor‐associated macrophages also exert an important role in ECM remodeling by secreting MMP‐2 and MMP‐9 [48]. Moreover, cancer cells can produce EVs to convey molecular information that may influence the function of distant NK cells, T cells, and antigen‐presenting cells to prepare an immunosuppressive environment in the future metastatic niche [49]. EVs display specific organotropism depending on their integrin expression profile [50]. However, even when CTCs find the optimal conditions for extravasation and the environment is favorable, a new tumor will not form automatically. Indeed, cancer cells (particularly breast cancer cells) can remain dormant for an extended period of time (i.e. tumor dormancy) [31]. Two different hypotheses have been proposed to explain this phenomenon. Dormant cancer cells may withdraw completely from the cell cycle (i.e. cancer cell dormancy) [51] or they continue to proliferate, but their number is counterbalanced by the number of cells that die (micro‐metastatic model) [51]. In the cancer cell dormancy model, cells can enter in a prolonged state of mitotic arrest in the G_0–1_ phase [52]. In the micro‐metastatic model, cells proliferate but the tumor does not grow in size. This control is intimately related to the process of neovascularization. Indeed, increasing the concentration of angiogenic agents (e.g. FGF or VEGF) promotes the tumor growth capacity by ‘waking up’ dormant cells [53]. When the microenvironment becomes favorable, cancer cells will leave dormancy and initiate a new metastasis. For instance, in mice, tobacco‐induced inflammation leads to the formation of neutrophil extracellular traps (NETs) that cleave ECM laminins. This cleavage generates a new epitope that activates the integrin signaling cascade in cancer cells, converting dormant cells into aggressive metastatic cells [54].

## 3 CTC interactions with blood cells during their journey

Following the detailed description of the various steps of the metastatic cascade, this new section briefly describes the interactions between CTCs and the surrounding cells in the blood compartment. The most aggressive CTCs can interact with cells in the blood compartment, such as platelets, immune cells, and CAFs. These interactions help CTCs to evade the immune system, and also facilitate adhesion, intravasation and invasion. To achieve this, CTCs skillfully use the blood cell plasticity to their advantage.

Cancer progression is strongly influenced by recruiting and activating platelets [55]. These anucleated cells, derived from the maturation of megakaryocytes in the bone marrow, are involved in thrombosis and hemostasis that occur also during inflammation, one of the hallmarks of cancer. TGF‐ß secretion by platelets and direct contact with cancer cells activate the TGF‐ß/SMAD and NF‐kB pathways [56]. These pathways are involved in the cancer cell invasive capacities and initiate their EMT. Platelets can also form clusters with CTCs in which CTCs are protected against the shear stress of the bloodstream [57], against NK cell‐mediated cytolysis [58] and against the immune system attacks. Platelets also facilitate CTC extravasation. Indeed, ATP secretion by tumor‐educated platelets activates the P2Y_2_ receptor, thus opening the transendothelial barrier and allowing CTC extravasation [59]. Taken together, these data demonstrate that platelets support CTCs on their journey.

CTCs interact with other cell types in the blood compartment. Neutrophils are an extremely abundant circulating white cell type and are also implicated in cancer development. Indeed, increased neutrophil number has been associated with poor prognosis. The association of CTCs with neutrophils in the bloodstream enhances their metastatic capacity by driving the cell cycle [60]. Furthermore, activated neutrophils can release DNA‐histone complexes and NETs that are involved in cancer progression [61]. NETs can sequester CTCs by binding to β1 integrins (expressed on the cancer cell surface), thereby promoting their dissemination [62]. Spiegel et al. [63] showed that neutrophils play a role in immune evasion by inhibiting NK cell function and also promote CTC extravasation by secreting IL‐1β and MMPs. Although the underlying mechanisms remain poorly understood, CTC interaction with neutrophils facilitates cancer cell dissemination.

Tumor‐associated macrophages are implicated in the primary tumor development, and also in CTC dissemination and extravasation. A study showed that when CTCs from small cell lung cancer were co‐cultured with peripheral blood mononuclear cells, they induced the differentiation of monocytes into macrophages to enhance angiogenesis, invasion, and immunosuppression [64]. Moreover, recent results suggest that the interaction between macrophages and CTCs promotes epithelial‐to‐mesenchymal plasticity and confers greater resistance to shear stress [65]. CTCs may fuse with macrophages to increase their chances of survival. The presence of these macrophage‐CTC fusions in the blood is a poor prognostic factor [66, 67]. When injected in mice, they can spread and colonize in distant sites [68]. The use of leukocyte and CTC markers to identify these macrophage‐CTC fusion events may be useful for predicting the prognosis of patients with ovarian cancer. A better understanding of the interactions between macrophages and CTCs would help to clarify the metastatic cascade.

MDSCs are a population of immature myeloid cells with immunosuppressive properties toward T lymphocytes, dendritic cells (DC) and NK cells. It has been hypothesized that clusters of CTCs with MDSCs form a defensive shield to avoid immune surveillance and to facilitate dissemination [69]. Sprouse et al. [70] demonstrated higher CTC dissemination in mice after co‐injection of CTCs (isolated from patients with melanoma or metastatic breast cancer) and polymorphonuclear‐MDSCs (PMN‐MDSCs) compared with injection of CTCs alone or PMN‐MDSCs alone. Co‐culture of CTCs with PMN‐MDSCs induced the pro‐tumor differentiation of PMN‐MDSCs via paracrine Nodal signaling, leading to increased production of reactive oxygen species [70] that can inhibit T cell differentiation and activation [71]. Indirectly, IL‐8 produced by cancer cells chemotactically attracts MDSCs and induces NET formation by MDSCs [72].

CAFs play an important role in tumor microenvironment remodeling and angiogenesis, but few data are available on their interactions with CTCs. CAF depletion in mice reduced the number of metastases because CTCs that travel with CAFs in the bloodstream display increased viability and colonization potential [73]. Moreover, through intercellular contacts and soluble factors, activated CAFs protect CTCs from shear stress [74]. Many evidences suggest that CAFs mediate CTC cluster formation [75].

All these studies show that CTCs do not travel alone in the bloodstream and that they adroitly subvert the functions of blood cells to increase their capacity for dissemination [76]. Nevertheless, little is known about the nature of these interactions.

## 4 How can CTCs evade immune cell attacks?

**Fig. 1:**
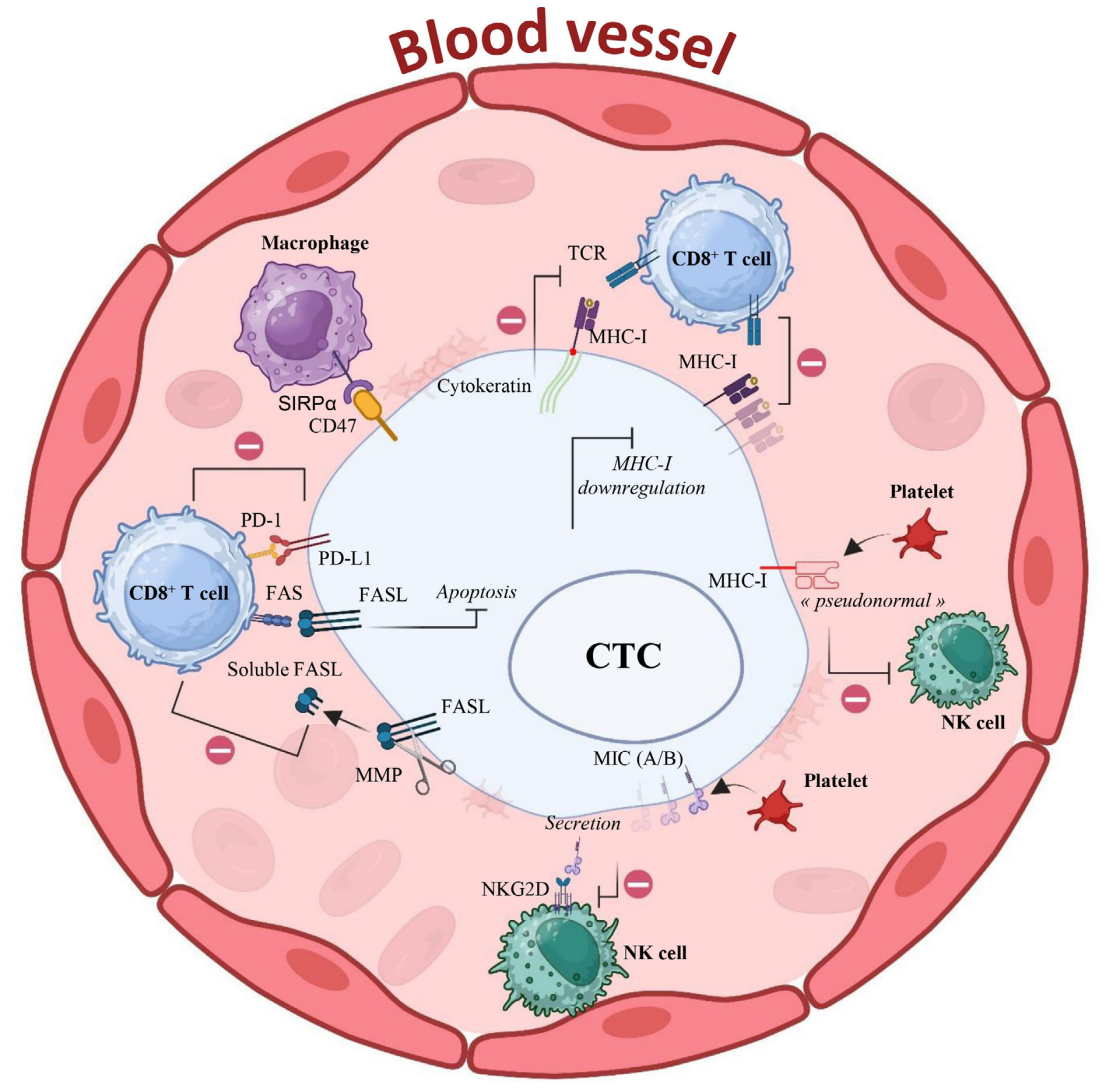
Immune evasion mechanisms of circulating tumor cells (CTCs). The schematic highlights the most important known mechanisms of immune evasion by CTCs. Through epigenetic and genetic mechanisms, the major histocompatibility complex I (MHC‐I) is downregulated in CTCs and this allows escaping identification by CD8^+^ T cells. Cytokeratin 8, 18 and 19 inhibit the interaction with the TCR expressed on CD8^+^ T cells. Platelets can confer a pseudonormal phenotype to the MHC‐I of CTCs, thus avoiding recognition by natural killer (NK) cells. Platelets also promote the downregulation of MIC A and MIC B, ligands of the NKG2D activating receptor. In addition, in CTCs, secretion of MIC A and MIC B is induced, promoting the protection against NK cell cytotoxic effects. Metalloproteases secreted by CTCs cleave the FAS receptor ligand (FASL), thus inhibiting the action of CD8^+^ T cells. FASL binding to the FAS receptor on CD8^+^ T cells inhibits CTC apoptosis. Expression of the immune checkpoint protein PD‐L1 maintains CD8^+^ T cells in an inactive state. Expression of CD47 on CTC surface sends a “don't eat me” signal by binding to the SIRPα receptor on macrophages. Created with BioRender.com. CTCs, circulating tumor cells; MHC, major histocompatibility complex; NK, natural killer; TCR, T‐cell receptor.

The immune system is a complex and dynamic network of different cell types that can discriminate between self and non‐self. Cancer cells are not totally considered non‐self and they sometimes escape the immune system. Moreover, immune cells can display both pro‐tumor and anti‐tumor activities. In the following section, we will discuss and propose molecular mechanisms of immune evasion by CTCs (Fig. 1; Table 1).

**Table 1:** Non‐exhaustive list of immune evasion mechanisms of CTCs and their analysis methods.

Mechanisms	Type of analysis	References
MHC‐I downregulation	Micrometastatic carcinoma CK18‐positive cells colabeled with monoclonal antibody W6/32 directed to a framework antigenic determinant of MHC class I heavy chains associated with β2‐microglobulin	[79]
MHC‐I « pseudonormal »	*In vitro* flow cytometry, immunofluorescent staining, confocal microscopy, and immunoelectron microscopy	[81]
MHC‐I dysfunction by cytokeratins expression	Co‐immunoprecipitation, combined with LC–tandem mass spectrometry and immunoblotting on carcinoma cell line derived from a subclavicular lymph node metastasis	[82]
MIC A/B underregulation	Flow cytometry, ELISA and RT‐PCR of tumors cells coincubated with platelets	[93]
CD47 upregulation	Gene expression analyses on CTCs from colorectal cancer	[85]
FasL expression	Flow cytometry on peripheral CD95 (FAS)‐positive T‐helper cells from CTC‐positive patients	[89]
PD‐L1 expression	CellSearch on metastatic breast cancer patients	[95]

Self‐recognition is an essential step in immune surveillance. Each cell is probed via the crosstalk between MHC‐I molecules expressed on its surface and the T cell receptors (TCR) on CD8^+^ T cells. The presence of a tumor‐associated antigen (TAA) bound to MHC‐I molecules is essential for the initiation of the immune response. CTCs exploit different mechanisms to escape this surveillance.

First, cancer cells can downregulate MHC‐I expression at their surface, thus hindering surveillance by cytotoxic T lymphocytes (CTLs) [77, 78]. It has also been reported that MHC‐I expression is frequently downregulated in micro‐metastatic carcinoma cells from bone marrow aspirates [79]. However, MHC‐I downregulation promotes NK cell cytotoxic function [80]. To avoid activation of CTLs and NK cells, CTCs must express MHC‐I but without TAA presentation. For instance, in *in vitro* experiments, platelets can confer to cancer cells in which MHC‐I is downregulated a non‐suspicious MHC‐I phenotype, known as “pseudonormal”, to avoid CTL and NK cell activation [81]. In addition, expression of cytokeratin (CK) 8 and its heterodimeric partners CK‐18 and CK‐19 by cancer cells can inhibit MHC‐I interaction with TCR on CTLs by binding to their activation domain [82]. This mechanism has been demonstrated using carcinoma cell lines derived from a subclavicular lymph node metastasis. CTCs also express CKs at their surface. Moreover, platelet aggregation to CTCs may promote loss of expression of MIC‐A and MIC‐B in CTCs (two ligands of the NKG2D receptor that is expressed on NK cells) [83].

Second, CTCs express some glycoproteins and receptors on their surface. The glycoprotein CD47 binds to its receptor signal‐regulatory protein α (SIRPα) that is expressed on the surface of DCs and macrophages and inhibits phagocytosis [84]. Genomic analyses in patients with colorectal cancer have shown that CTCs overexpress CD47, thus inducing a “don't eat me” signal [85]. CD47‐expressing CTCs have also been found in patients with metastatic non‐small cell lung cancer and are associated with poor progression‐free survival [86]. CD47 expression on the CTC surface has also been reported in metastatic head and neck squamous cell carcinoma [87] and metastatic breast cancer [88]. Moreover, sometimes, CD47 expression on CTCs and on the original tumor can show a lack of concordance [88].

Third, to avoid lysis induced by CTLs, CTCs may express ligands, such as FASL [89]. In primary breast cancer, a flow cytometry study showed that CTC‐positive patients have more FAS‐positive T cells [89]. Binding of FASL to the FAS transmembrane receptor expressed on the surface of CD8^+^ T cells initiates apoptosis [90]. Moreover, autocrine secretion of soluble FASL may constitute an additional mechanism of escape from T‐cell surveillance [91]. Although this mechanism is poorly understood and not explicitly proven for CTCs, there is some evidence of the FAS/FASL pathway implication in the immune escape by CTCs.

CTCs can also keep CD8^+^ T cells in an inactive state by expressing immune checkpoint proteins. Much current research is focusing on these interactions, and programmed cell death protein 1 (PD‐1) and its ligand (PD‐L1) perfectly illustrate the phenomenon. The interaction between PD‐1 and PD‐L1 maintains immune tolerance by reducing cytokine production and suppressing T‐cell proliferation [92]. CTCs from various cancer types can express PD‐L1 [93, 94, 95] and this is generally considered a poor prognostic marker [93]. Moreover, PD‐L1 expression heterogeneity in CTCs, compared to the primary tumor, may confer an advantage regarding immune evasion [15]. This non‐exhaustive review of possible immune evasion mechanisms in CTCs illustrates the variety of mechanisms involved, but also our limited knowledge.

## 5 Immunotherapy in patients with cancer

Today, immunotherapy is considered a therapeutic revolution in cancer treatment. However, patient response to treatment is variable and the underlying reasons are still elusive. Several classes of immunotherapy molecules are emerging for cancer treatment, such as monoclonal antibodies against immune checkpoint factors, vaccines and adoptive T cell therapy.

Monoclonal antibodies against immune checkpoint components inhibit cancer cell‐induced immunosuppression. By expressing some ligands on their surface, such as cytotoxic T‐lymphocyte‐associated antigen 4 (CTLA‐4) or PD‐L1, cancer cells can maintain T cells in an inactive state. PD‐L1 is often overexpressed on cancer cells and in the tumor microenvironment. The US Food and Drug Administration (FDA) has approved some immune checkpoint inhibitors (ICIs) against PD‐L1, such as pembrolizumab and nivolumab, and against CTLA‐4, such as ipilimumab. Pembrolizumab and nivolumab are currently indicated for head and neck squamous cell carcinoma, melanoma, non‐small cell lung cancer, and Hodgkin's lymphoma. Ipilimumab was approved for melanoma in 2011 [96]. However, many patients will not respond to ICIs. For instance, pembrolizumab (against PD‐L1) monotherapy shows response rates of ≥35% in patients with advanced gastric/gastroesophageal junction cancer [97], advanced small‐bowel adenocarcinoma [98], advanced melanoma [99], advanced cervical squamous cell cancer [100] and advanced non‐clear cell renal cell carcinoma [101]. Similarly, nivolumab (against PD‐L1) displays response rates of almost 30% in patients with advanced non‐small cell lung cancer [102], metastatic urothelial carcinoma [103] and advanced hepatocellular carcinoma [104]. Ipilimumab (against CTLA 4) shows even lower response rates: ~ 20% in advanced melanoma [105, 106]. Cancer cells often acquire mutations that inactivate the JAK1 and JAK2 pathways, thus partly explaining resistance to anti‐PD‐1 therapies [107]. Moreover, the human leukocyte antigen (HLA)‐I genotype has a major influence on the response rates to ICIs [108]. Although most patients do not respond to these immunotherapies, therapeutic antibodies against PD‐1, PD‐L1 or CTLA4 have markedly increased progression‐free survival and overall survival in various cancers, especially when combined with chemotherapy [109, 110, 111, 112].

Cancer vaccines involve injecting tumor antigens combined with an adjuvant to stimulate DCs. These vaccines can be based on peptides, RNA, DNA, or even directly matured DCs. The aim is to stimulate the patients' adaptive immunity to prevent tumor development. The efficacy of these vaccines relies on the strong activation of CD4^+^ and CD8^+^ T cells, and their infiltration into the tumor. However, many patients do not respond or develop resistance to anti‐tumor vaccines. Loss of HLA class 1 expression is one of the main mechanisms of resistance to therapeutic vaccines by hiding tumor antigens [113]. The tumor microenvironment also contributes to resistance mechanisms, notably by interfering with T‐cell activation and by secreting immunosuppressive cytokines [114, 115]. This leads to low response rates, particularly in patients with melanoma (2.6%) [116]. In the meta‐analysis by Nagorsen and Eckhard, the response rate to therapeutic vaccine‐based immunotherapies for colorectal cancer was 0.9% [117]. More recently, the combination of the mRNA‐based vaccine RO7198457 with the anti‐PD‐L1 monoclonal antibody atezolizumab has shown a response rate up to 30% in patients with advanced solid cancers [118], a result comparable to that obtained with the antibody alone. Similarly, the combination of the mRNA‐based vaccine 4157 and pembrolizumab showed a 30% response rate in patients with advanced or metastatic cancer [119]. These data suggest that combining these therapeutic vaccines with ICIs does not increase the response rate compared with ICIs alone.

Immunotherapies also include the injection of immunoactivating cytokines, such as IL‐2, leading to T‐cell activation and proliferation. In patients with melanoma, these therapies show a response rate of 12% as monotherapy and 16% in combination with vaccines [120]. Similarly, the combination of IL‐2 with the peptide‐based gp100 vaccine displays a response rate of 16% [121].

The aim of these approaches is to induce the anti‐cancer immune response, but there are also immunotherapies with direct anti‐cancer properties. The transfer of genetically modified T cells, expanded *ex‐vivo* to target specific tumor antigens in the patient, is an important area of immunotherapy research. The development of CAR‐T cells represents one of the most promising approaches in this field. Its success has been widely confirmed in patients with hematological cancers [122, 123, 124], but remains to be validated in solid cancers. In a meta‐analysis of 22 studies published in 2018, the response rate to CAR‐T cell therapies was 9% in solid tumors [125]. The administration of CAR‐T cells in 17 patients with HER2‐positive sarcomas led to a positive response in 4 patients and in 3 of them the tumor could be removed [126]. One explanation for these low response rates is that tumors acquire mutations leading to loss of expression of the CAR‐T cell targets [127]. New bispecific CAR‐T cells have been developed to limit this immune escape [128]. Overall, the tumor microenvironment is the main barrier to the efficacy of CAR‐T cells in solid tumors. Moreover, administration of CAR‐T cells is associated with high toxicity: cytokine‐release syndrome, neurotoxicity, off‐targeted effects, and acute respiratory distress syndrome [129].

Today, new classes of antibodies are undergoing rapid preclinical and clinical development. For instance, by targeting different antigens, bispecific antibodies offer the possibility of assisting and promoting tumor cell destruction by the immune synapse [130]. In 2022, tebentafusp was approved for the treatment of metastatic uveal melanoma. This bispecific antibody targets the gp100 peptide exposed on the surface of HLA‐A*02:01 and CD3 [131]. In a phase 3 clinical trial involving 378 patients, tebentafusp showed greater progression‐free survival (31% vs. 19%) than conventional treatments (pembrolizumab, ipilimumab, or dacarbazine) [132] and also longer overall survival [132]. In 2022, the European Medicines Agency (EMA) granted market authorization for the bispecific antibody teclistamab for relapsed/refractory multiple myeloma. It targets both the CD3 receptor on T cells and the membrane protein B‐cell maturation antigen overexpressed on myeloma cells. In 165 patients with myeloma refractory to three lines of therapy enrolled in a phase 1–2 clinical trial, the overall response rate to teclistamab was 63%, and the mean progression‐free survival was 11.3 months [133]. Today, bispecific antibodies offer broader prospects, and many of them are currently in clinical trials for both solid and hematological cancers.

In conclusion, immunotherapies face many challenges and the response rates rarely exceed 30%. Combining these therapies with chemotherapy or radiotherapy increases these response rates, but the discovery of immune evasion mechanisms remains a key factor for predicting and improving the efficacy of immunotherapies.

## 6 CTC challenges

CTCs are among the most aggressive cancer cells. They are the key players in the metastatic cascade, but little is known about their immune evasion mechanisms. Their interactions with different cell types in the blood compartment often enable them to increase their chances of survival. Targeting CTCs during the different steps of the metastatic cascade could contribute to limit metastasis formation. Therefore, it is essential to identify the mechanisms of CTC immune escape. As most of the known mechanisms involve interactions of proteins expressed on the cell surface (e.g. CD47, PD‐L1) and some are regulated by various post‐translational modifications (PTMs), genomic tools are not sufficient to identify new immune escape mechanisms.

Several groups have already performed global proteomic analyses of CTCs using different methodological approaches. Sinkala et al. [134] used a microfluidic western blot method for a multiplex protein panel to analyze single CTCs from patients with estrogen receptor‐positive breast cancer. Moreover, proteomic profiling of 1–5 CTCs allowed defining a panel of prostate cancer‐specific proteins and discriminating between CTCs and white blood cells [135]. In head and neck squamous cell carcinoma, proteomic analysis of CTCs by high‐dimensional single‐cell mass cytometry allowed the identification of common CTC subgroups. Their proteomic profiles could be exploited to develop new biomarkers and for patient stratification for immune checkpoint inhibitor treatment [136]. Proteomic analysis by MS has been used to analyze CTCs from mice xenografted with breast cancer cells [137].

Nevertheless, a key step in assessing CTC heterogeneity using omic methods is to increase their number because they are extremely rare in the blood compartment. The culture and expansion of CTCs *in vitro* provides quantitative (a large number of CTCs) and qualitative (metastasis‐initiating CTCs) added value [138]. In addition, to overcome the low CTC availability in blood samples, it is possible to increase the volume of blood collected by leukapheresis. In this way, liters of blood can be screened [139]. The identification of new mechanisms of CTC immune evasion will require the proteomic study of their surface, opening a new and promising avenue for the discovery of new immunotherapies.

## 7 Proteomic tools to assess CTC surfaceome

### 7.1 Immunopeptidomics

Research in CTC biology, immune evasion mechanisms and therapeutics targets has long been hampered by the rarity of these events and the fact that the only FDA‐approved system, the CellSearch® system, uses fixed cells. Few permanent CTC lines have been established for colon, breast and lung cancer, paving the way for new approaches [140, 141]. These CTC lines represent a rare source of metastasis‐initiating cells. These cells, which have the potential to self‐renew and expand *in vitro*, have all the properties needed to develop secondary tumors in patients. Proteomic tools can be very useful to study the surfaceome of these CTCs in order to understand their escape mechanisms and to develop new treatments.

**Fig. 2:**
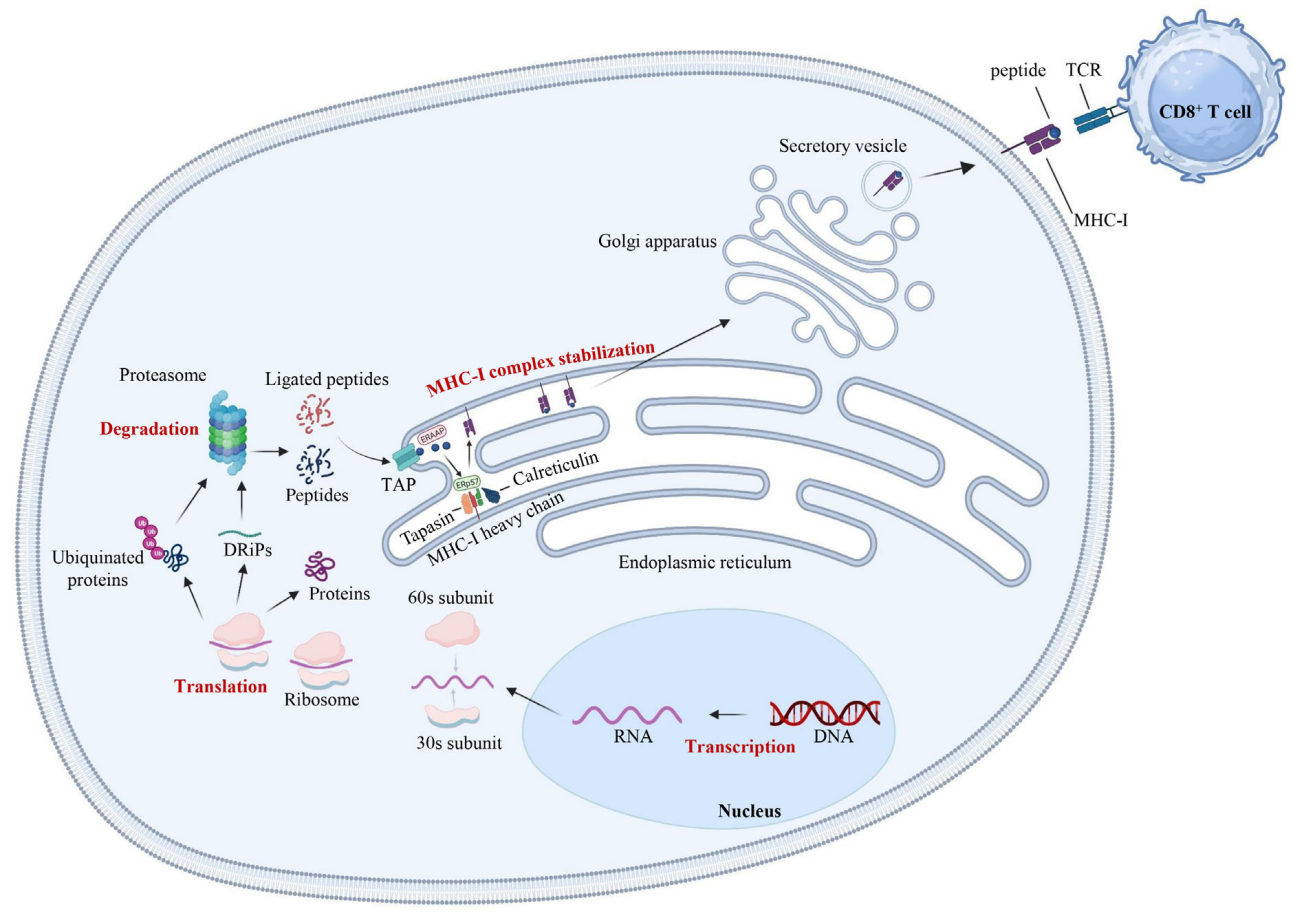
MHC class I antigen presentation pathway. Peptide presentation on the major histocompatibility complex I (MHC‐I) surface is a multi‐step process. After transcription, the RNA of a given protein is taken up by the 30S and 60S ribosomal subunits to initiate translation. Errors in translation lead to the formation of defective ribosomal products (DRiPs). Unfolded or inactive proteins are ubiquitinated and sent to the proteasome where they are degraded into peptides. The proteasome also has a non‐genetically encoded ligation activity that leads to ligated peptides. Peptides are then translocated into the lumen of the endoplasmic reticulum (ER) by a transporter associated with antigen presentation (TAP). MHC‐I molecules are stabilized and conformed by chaperone proteins, such as tapasin, calreticulin and ERp57. Peptides without affinity for MHC‐I molecules are taken up by ER aminopeptidase associated with antigen processing (ERAAP). Once the peptide is bound to MHC‐I molecules, the complex is delivered to the membrane to be recognized by the TCR of CD8^+^ T cells. Created with BioRender.com. DRiPs, defective ribosomal products; ERAAP, ER aminopeptidase associated with antigen processing; MHC, major histocompatibility complex; TAP, transporter associated with antigen presentation; TCR, T‐cell receptor.

Immunopeptidomics defines the study of the immunopeptidome (i.e. the peptides presented by the MHC molecules) mainly using LC and MS‐based methods [142]. This has already allowed obtaining major insights into the biosynthesis of peptides, right up to their binding to HLA molecules, that will be briefly summarized here. Peptide presentation on the surface of individual human cells is a dynamic and constant process and provides a picture of the cell health state [143]. When a cell is infected or had undergone transformation, its immunopeptidome profile changes and this is sensed by the immune system. Peptides are presented to the immune system using two pathways: (a) The antigen processing and presentation machinery pathway degrades intracellular ubiquitinated proteins via the nuclear and cytosolic proteasomes. Then, the short peptides (8–15 amino acids) are taken to the endoplasmic reticulum via transporter associated with antigen processing (TAP) molecules, where they bind to HLA class I molecules (Fig. 2). These complexes are then exported to the cell surface to be scanned by the TCR expressed on CD8^+^ T cells [144]. (b) The second pathway for peptide presentation on the cell surface involves the degradation of endocytic extracellular proteins in the endosome‐lysosome compartment. These peptides (up to 25 amino acids) are then loaded onto HLA class II molecules and exported to the cell surface. Surface presentation of these complexes to CD4^+^ T cells is exclusively done by professional antigen‐presenting cells, including DCs, B cells, macrophages and thymic epithelial cells [145]. In cancer cells, the presented peptides (neo‐peptides or neo‐antigens) may be the result of various gene alterations, such as non‐synonymous mutations that modify the protein sequence, gene fusions leading to fusion proteins, cancer‐specific alternative mRNA splicing or aberrant PTMs [144].

Therefore, the immunopeptidome is a modulator of the immune response, and understanding how peptides are edited is fundamental for the analysis of the interactions between cancer and immune cells. Peptide editing is regulated by numerous PTMs through enzymes that can be deregulated in cancer. These PTMs can lead to tumor‐specific immunogenic peptides. Phosphorylation, glycosylation and citrullination are the most widely studied PTMs. In cancer cells, MHC‐I and MHC‐II molecules can present phosphorylated peptides [146, 147] that activate CD8^+^ and CD4^+^ T cells [148, 149]. The conversion of an arginine residue to citrulline (citrullination) by deamination is mediated by protein arginine deiminase (PADI) family members [150, 151]. Some studies suggest that the citrullinome is a rich source of cancer neo‐antigens that are involved in immune response [151]. Citrullinated peptides are predominantly presented by MHC‐II molecules via autophagy that is induced by tumorigenesis [152]. Then, these peptides can stimulate the Th1 response [152]. Therefore, neo‐antigens are therapeutic candidates to boost the anti‐tumor immune response [153, 154]. In addition, PTMs of peptides presented by the MHC‐I and ‐II molecules are widely implicated in anti‐tumor immune recognition, but only few of these PTMs have been studied. In the case of CTCs, no immunopeptidome data is available yet.

Given that the immunopeptiome is involved in immune recognition, its analysis is implicated in the development of immunotherapies. Proteomic tools are used to identify TAAs and tumor‐specific antigens (TSAs) that can be targeted by immunotherapies [155, 156]. TSAs are ideal targets because they are not expressed on healthy cells. There are many sources of TSAs: cancer‐induced aberrant PTMs, cryptic peptides, variants resulting from the use of a non‐canonical open reading frame or a reading frame shift, endogenous retroelements, and defective ribosome products [156]. Strategies to identify these peptides are based first on the analysis of next‐generation sequencing data, including exome sequencing, RNA sequencing and ribosome sequencing. Ribosome profiling defines the mRNA regions that are translated into proteins. Then, many bioinformatic tools are available to predict whether a given peptide is likely to be present on the surface. These tools are generally based on the probability of degradation by the proteasome or the affinity between peptide and HLA molecules and are used to refine the analysis [157, 158, 159, 160]. Then, HLA‐peptide complexes are isolated by immunoprecipitation using specific antibodies and identified by MS. MS data are compared with protein databases or customized databases using computational searching engines, such as MaxQuant [161]. This strategy for identifying TSAs associated with an immune response is at the basis of the design of innovative therapies, such as peptide‐based vaccines and CAR‐T cells [162, 163, 164].

Today, MS is the only unbiased method for identifying peptides expressed on the surface of MHC‐I and II molecules. The majority of immunopeptidomic studies use data‐dependent tandem MS (MS/MS) acquisition (DDA). Although this method is considered ideal for new target discovery, it lacks the sensitivity to detect less abundant ions and displays low reproducibility [164]. Pak et al. [165] demonstrated that using the data‐independent acquisition (DIA) method for immunopeptidomic analyses could improve neo‐antigen detection.

Combined with next‐generation sequencing methods and prediction algorithms, MS offers a powerful tool to identify immune response mechanisms in the context of cancer. Specifically, this strategy and the few available CTC lines [141] could be used for immunopeptidomic analyses to bring needed insights into the HLA‐peptide complexes and profiles in CTCs.

### 7.2 Surface glycoproteomics

Glycosylation is a key cellular PTM that regulates a wide range of pathophysiological functions. This PTM involves the addition of monosaccharides and complex carbohydrates to proteins through the coordinated action of many different substrate‐dependent enzymes in the endoplasmic reticulum and Golgi apparatus. N‐linked glycosylation involves the addition of glycans to the nitrogen of asparagine residues, while O‐linked glycosylation targets the oxygen atoms of serine and threonine residues. Most glycoproteins are secreted or sent to the surface. Several studies have shown that deregulation of the glycosylation machinery is associated with tumor development [166, 167, 168]. Aberrant or incomplete glycosylation due to overexpression or deregulation of transferases affects cell adhesion to the basal membrane, cell–cell interactions, ECM remodeling, cell proliferation, genome stability and interaction with immune cells [168].

Glycoproteomics describes the system‐wide study of the glycosylation profile, glycan structures and modification sites using mainly LC/MS methods to assign a biological function to glycoproteins [22]. The workflow of a glycoproteomic analysis includes several key steps: sample selection, processing, enzymatic digestion, enrichment and MS analysis. Lectins are involved in various biological functions, such as immune defense and cell–cell interactions, and are considered carbohydrate binders [169]. Therefore, lectins are sometimes used to separate glycoproteins from other proteins in LC–MS approaches or to analyze different profiles. Analytical methods using lectins include multi‐lectin affinity chromatography [170], lectin microarray [171] and enzyme‐linked lectin assays [172]. The analysis of glycoproteins expressed on the cancer cell surface appears to be relevant for the discovery of new immune evasion mechanisms because some PTMs may be at the core of interactions with the immune system and CTCs.

**Fig. 3:**
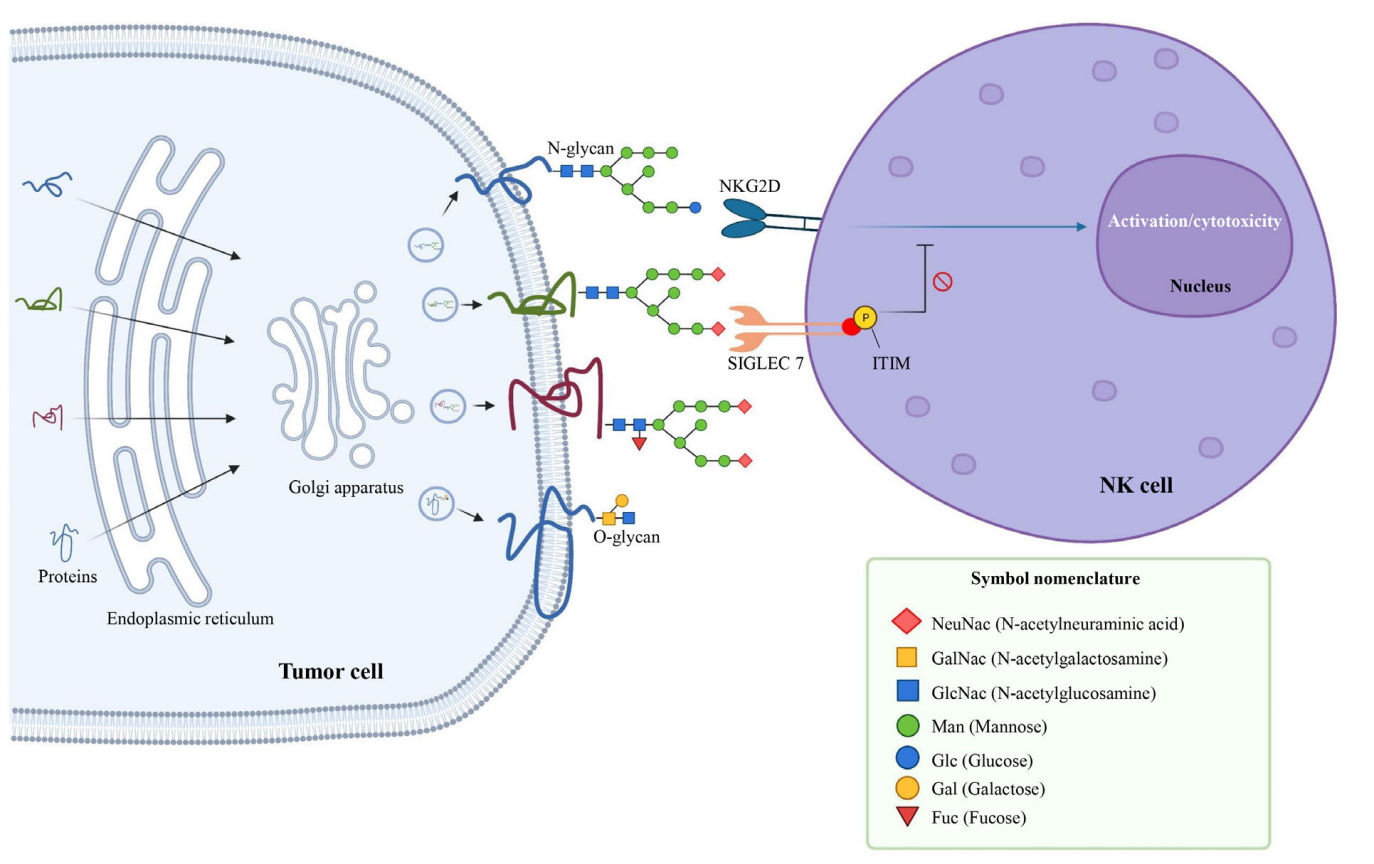
Glycosylation to avoid natural killer (NK) cell cytotoxicity. Protein glycosylation begins in the endoplasmic reticulum. It involves the addition of carbohydrates to polypeptide chains by glycosyltransferases and oligosaccharyltransferases. This process is deregulated in cancer, and this often leads to the aberrant addition of sialic acid at the end of the chain. The most common sialic acid in humans is N‐acetylneuraminic acid (NeuNAc). Sialic acid molecules bind to the sialic acid‐binding immunoglobulin‐like lectin 7 (SIGLEC‐7) receptor on NK cells. Binding induces phosphorylation of the SIGLEC‐7 immunoreceptor tyrosine‐based inhibitory motif (ITIM). Upon phosphorylation, this domain can recruit phosphatases (SHP1/2) that block the NKG2D activation pathway, thus preventing NK cell activation. Created with BioRender.com. ITIM, immunoreceptor tyrosine‐based inhibitory motif; NK, natural killer; SIGLEC, sialic acid‐binding immunoglobulin‐like lectin.

The addition of sialic acid by sialyltransferases at the end of glycans is a PTM often deregulated in breast [173], pancreatic [174], ovarian [175] and lung cancer [176]. Sialic acid plays a pivotal role in immunomodulation and immune evasion of cancer cells (Fig. 3). Sialylated proteins are recognized by sialic acid‐binding immunoglobulin‐type lectins (SIGLEC) expressed on the surface of immune cells [177]. Specifically, SIGLEC‐7 and ‐9 are expressed on the surface of DCs, NK cells, CD8^+^ T cells, and monocytes. These interactions promote immunosuppression and protect cancer cells. Binding of SIGLEC‐7 and ‐9 to their ligands on pancreatic ductal adenocarcinoma (PDAC) cells directs the differentiation of monocytes into macrophages with an immunosuppressive phenotype [177]. Recently, *SIGLEC9* mRNA expression was correlated with poor prognosis and high infiltration of immune cells in various cancer types, such as adrenocortical carcinoma, lung adenocarcinoma, colon adenocarcinoma, glioblastoma multiforme and prostate adenocarcinoma [178]. Läubli et al. [179] showed that blocking SIGLEC‐9 expressed on the surface of myelomonocytic cells enhanced the neutrophil anti‐tumor activity. Additionally, they found that immune surveillance was improved in mice deficient in Siglec‐E, the murine equivalent of SIGLEC‐9 [179]. Furthermore, overexpression of sialylated glycans on the surface of B16 melanoma cells affects the balance between effector and regulatory T cells (Treg), facilitating the presence of Treg cells and thus orchestrating immune escape [180]. Sialoglycan interaction with SIGLEC‐7 and 9 expressed on the NK cell surface reduces their cytotoxicity [181]. This mechanism allows cancer cells in which MHC‐I has been downregulated to escape NK cell‐mediated lysis.

The glycosylation profile of cancer cell surface proteins greatly influences the invasion process, notably by modulating their interactions with immune cells. The multifactorial modifications leading to abnormal glycosylation have never been investigated in CTCs, and glycoproteomic studies could provide insights into the different mechanisms used by CTCs to bypass the circulating immune system. Some groups are developing tools for proteomic analysis of single cells isolated from blood samples. The current proteomic methods allow working with a reduced number of cells. For instance, the LTQ Orbitrap Velos Pro Mass Spectrometer allowed differentiating between CTCs and lymphocytes in blood samples, and to categorize CTCs from different cancer types in function of their profiles [182]. Proteomic methods could be powerful tools to discover new biomarkers and potentially new biological mechanisms.

## 8 Conclusions

Immunotherapies have revolutionized cancer treatment, but there are still too many patients who do not respond to these therapies. Cancer cells develop resistance or simply do not express the therapeutic targets. Moreover, there is no targeted therapy against CTCs and particularly metastasis‐initiating CTCs. Yet, these are the most aggressive tumor cells and they are thought to be implicated in the immune evasion mechanisms and therapy resistance due to their phenotypical heterogeneity. The available few CTC lines and proteomic tools, such as immunopeptidomic and glycoproteomic assays, could provide the means for an in‐depth analysis of the various mechanisms used by CTCs to evade the immune system and targeted therapies. These emerging and innovative tools combined with these metastasis‐initiator CTC lines could give a unique chance to pave the way for understanding CTC immune evasion properties and for the development of therapies targeting specific neo‐antigens expressed by CTCs.

## Conflict of interest

The authors declare no conflict of interest.

## Author contributions

DM (Conceptualization‐Equal, Investigation‐Lead, Methodology‐Equal, Validation‐Equal, Writing—original draft‐Lead, Writing—review & editing‐Lead), JV (Validation‐Equal, Writing—original draft‐Equal, Writing—review & editing‐Equal), CH (Validation‐Equal, Writing—original draft‐Equal, Writing—review & editing‐Equal), and CAP (Conceptualization‐Equal, Methodology‐Equal, Supervision‐Lead, Validation‐Equal, Writing—original draft‐Equal, Writing—review & editing‐Equal).

## References

[1] Hanahan D , Weinberg RA . Hallmarks of cancer: the next generation. Cell. 2011;144(5):646–674. 21376230 10.1016/j.cell.2011.02.013

[2] Chambers AF , Groom AC , MacDonald IC . Dissemination and growth of cancer cells in metastatic sites. Nat Rev Cancer. 2002;2(8):563–572. 12154349 10.1038/nrc865

[3] Nguyen DX , Bos PD , Massagué J . Metastasis: from dissemination to organ‐specific colonization. Nat Rev Cancer. 2009;9(4):274–284. 19308067 10.1038/nrc2622

[4] Pantel K , Speicher M . The biology of circulating tumor cells. Oncogene. 2016;35(10):1216–1224. 26050619 10.1038/onc.2015.192

[5] Follain G , Herrmann D , Harlepp S , Hyenne V , Osmani N , Warren SC , et al. Fluids and their mechanics in tumour transit: shaping metastasis. Nat Rev Cancer. 2020;20(2):107–124. 31780785 10.1038/s41568-019-0221-x

[6] Strilic B , Offermanns S . Intravascular survival and extravasation of tumor cells. Cancer Cell. 2017;32(3):282–293. 28898694 10.1016/j.ccell.2017.07.001

[7] Cristofanilli M . Circulating tumor cells, disease progression, and survival in metastatic breast cancer. Semin Oncol. 2006;33:9–14. 10.1053/j.seminoncol.2006.03.016 16797376

[8] Pantel K , Alix‐Panabières C . Circulating tumour cells in cancer patients: challenges and perspectives. Trends Mol Med. 2010;16(9):398–406. 20667783 10.1016/j.molmed.2010.07.001

[9] Schwarzenbach H , Hoon DS , Pantel K . Cell‐free nucleic acids as biomarkers in cancer patients. Nat Rev Cancer. 2011;11(6):426–437. 21562580 10.1038/nrc3066

[10] Kalluri R , LeBleu VS . The biology, function, and biomedical applications of exosomes. Science. 2020;367(6478):eaau6977. 32029601 10.1126/science.aau6977 PMC7717626

[11] Best MG , Sol N , In 't Veld SGJG , Vancura A , Muller M , Niemeijer ALN , et al. Swarm intelligence‐enhanced detection of non‐small‐cell lung cancer using tumor‐educated platelets. Cancer Cell. 2017;32(2):238–252.e9. 28810146 10.1016/j.ccell.2017.07.004 PMC6381325

[12] Anfossi S , Babayan A , Pantel K , Calin GA . Clinical utility of circulating non‐coding RNAs—an update. Nat Rev Clin Oncol. 2018;15(9):541–563. 29784926 10.1038/s41571-018-0035-x

[13] Alix‐Panabières C , Pantel K . Liquid biopsy: from discovery to clinical application. Cancer Discov. 2021;11(4):858–873. 33811121 10.1158/2159-8290.CD-20-1311

[14] Toss A , Mu Z , Fernandez S , Cristofanilli M . CTC enumeration and characterization: moving toward personalized medicine. Ann Transl Med. 2014;2(11):108. 25489582 10.3978/j.issn.2305-5839.2014.09.06 PMC4245508

[15] Jacot W , Mazel M , Mollevi C , Pouderoux S , D'Hondt V , Cayrefourcq L , et al. Clinical correlations of programmed cell death ligand 1 status in liquid and standard biopsies in breast cancer. Clin Chem. 2020;66(8):1093–1101. 32712650 10.1093/clinchem/hvaa121

[16] Yewdell JW . MHC class I immunopeptidome: past, present, and future. Mol Cell Proteomics. 2022;21(7):100230. 35395404 10.1016/j.mcpro.2022.100230 PMC9243166

[17] León‐Letelier RA , Katayama H , Hanash S . Mining the immunopeptidome for antigenic peptides in cancer. Cancer. 2022;14(20):4968. 10.3390/cancers14204968 PMC9599891 36291752

[18] Zhang X , Qi Y , Zhang Q , Liu W . Application of mass spectrometry‐based MHC immunopeptidome profiling in neoantigen identification for tumor immunotherapy. Biomed Pharmacother. 2019;120:109542. 31629254 10.1016/j.biopha.2019.109542

[19] Thomas D , Rathinavel AK , Radhakrishnan P . Altered glycosylation in cancer: a promising target for biomarkers and therapeutics. Biochim Biophys Acta Rev Cancer. 2021;1875(1):188464. 33157161 10.1016/j.bbcan.2020.188464 PMC7855613

[20] Kailemia MJ , Park D , Lebrilla CB . Glycans and glycoproteins as specific biomarkers for cancer. Anal Bioanal Chem. 2017;409:395–410. 27590322 10.1007/s00216-016-9880-6 PMC5203967

[21] Wen R , Zhao H , Zhang D , Chiu CL , Brooks JD . Sialylated glycoproteins as biomarkers and drivers of progression in prostate cancer. Carbohydr Res. 2022;519:108598. 35691122 10.1016/j.carres.2022.108598

[22] Bagdonaite I , Malaker SA , Polasky DA , Riley NM , Schjoldager K , Vakhrushev SY , et al. Glycoproteomics. Nat Rev Methods Primers. 2022;2(1):48.

[23] Ashworth T . A case of cancer in which cells similar to those in the tumours were seen in the blood after death. Aust Med J. 1869;14:146.

[24] Seyfried TN , Huysentruyt LC . On the origin of cancer metastasis. Crit Rev Oncog. 2013;18(1–2):18. 10.1615/critrevoncog.v18.i1-2.40 PMC3597235 23237552

[25] Eslami‐S Z , Cortés‐Hernández LE , Alix‐Panabières C . The metastatic cascade as the basis for liquid biopsy development. Front Oncol. 2020;10:1055. 32850309 10.3389/fonc.2020.01055 PMC7396546

[26] Obenauf AC , Massagué J . Surviving at a distance: organ‐specific metastasis. Trends Cancer. 2015;1(1):76–91. 28741564 10.1016/j.trecan.2015.07.009 PMC4673677

[27] Massagué J , Batlle E , Gomis RR . Understanding the molecular mechanisms driving metastasis. Mol Oncol. 2017;11(1):3–4. 28085221 10.1002/1878-0261.12024 PMC5423223

[28] Drake JM , Strohbehn G , Bair TB , Moreland JG , Henry MD . ZEB1 enhances transendothelial migration and represses the epithelial phenotype of prostate cancer cells. Mol Biol Cell. 2009;20(8):2207–2217. 19225155 10.1091/mbc.E08-10-1076 PMC2669028

[29] Sun T , Zhao N , Zhao XL , Gu Q , Zhang SW , Che N , et al. Expression and functional significance of Twist1 in hepatocellular carcinoma: its role in vasculogenic mimicry. Hepatology. 2010;51(2):545–556. 19957372 10.1002/hep.23311

[30] Allard WJ , Matera J , Miller MC , Repollet M , Connelly MC , Rao C , et al. Tumor cells circulate in the peripheral blood of all major carcinomas but not in healthy subjects or patients with nonmalignant diseases. Clin Cancer Res. 2004;10(20):6897–6904. 15501967 10.1158/1078-0432.CCR-04-0378

[31] Meng S , Tripathy D , Frenkel EP , Shete S , Naftalis EZ , Huth JF , et al. Circulating tumor cells in patients with breast cancer dormancy. Clin Cancer Res. 2004;10(24):8152–8162. 15623589 10.1158/1078-0432.CCR-04-1110

[32] Aceto N , Bardia A , Miyamoto DT , Donaldson MC , Wittner BS , Spencer JA , et al. Circulating tumor cell clusters are oligoclonal precursors of breast cancer metastasis. Cell. 2014;158(5):1110–1122. 25171411 10.1016/j.cell.2014.07.013 PMC4149753

[33] Liotta LA , Stetler‐Stevenson WG . Tumor invasion and metastasis: an imbalance of positive and negative regulation. Cancer Res. 1991;51(18_Suppl):5054s–5059s. 1884381

[34] Hong Y , Fang F , Zhang Q . Circulating tumor cell clusters: what we know and what we expect. Int J Oncol. 2016;49(6):2206–2216. 27779656 10.3892/ijo.2016.3747 PMC5117994

[35] Sharma D , Brummel‐Ziedins KE , Bouchard BA , Holmes CE . Platelets in tumor progression: a host factor that offers multiple potential targets in the treatment of cancer. J Cell Physiol. 2014;229(8):1005–1015. 24374897 10.1002/jcp.24539

[36] Maeshiro M , Shinriki S , Liu R , Nakachi Y , Komohara Y , Fujiwara Y , et al. Colonization of distant organs by tumor cells generating circulating homotypic clusters adaptive to fluid shear stress. Sci Rep. 2021;11(1):6150. 33731803 10.1038/s41598-021-85743-z PMC7969766

[37] Cheung KJ , Padmanaban V , Silvestri V , Schipper K , Cohen JD , Fairchild AN , et al. Polyclonal breast cancer metastases arise from collective dissemination of keratin 14‐expressing tumor cell clusters. Proc Natl Acad Sci USA. 2016;113(7):E854–E863. 26831077 10.1073/pnas.1508541113 PMC4763783

[38] Krol I , Schwab FD , Carbone R , Ritter M , Picocci S , de Marni ML , et al. Detection of clustered circulating tumour cells in early breast cancer. Br J Cancer. 2021;125(1):23–27. 33762721 10.1038/s41416-021-01327-8 PMC8257701

[39] Kurma K , Alix‐Panabières C . Mechanobiology and survival strategies of circulating tumor cells: a process towards the invasive and metastatic phenotype. Front Cell Dev Biol. 2023;11:1188499. 37215087 10.3389/fcell.2023.1188499 PMC10196185

[40] Paget S . The distribution of secondary growths in cancer of the breast. Lancet. 1889;133(3421):571–573. 2673568

[41] Welch DR , Hurst DR . Defining the hallmarks of metastasis. Cancer Res. 2019;79(12):3011–3027. 31053634 10.1158/0008-5472.CAN-19-0458 PMC6571042

[42] Brodt P , Fallavollita L , Bresalier RS , Meterissian S , Norton CR , Wolitzky BA . Liver endothelial E‐selectin mediates carcinoma cell adhesion and promotes liver metastasis. Int J Cancer. 1997;71(4):612–619. 9178816 10.1002/(sici)1097-0215(19970516)71:4<612::aid-ijc17>3.0.co;2-d

[43] Kannagi R , Izawa M , Koike T , Miyazaki K , Kimura N . Carbohydrate‐mediated cell adhesion in cancer metastasis and angiogenesis. Cancer Sci. 2004;95(5):377–384. 15132763 10.1111/j.1349-7006.2004.tb03219.x PMC11159147

[44] Fukuda M , Hiraoka N , Yeh J‐C . C‐type lectins and sialyl Lewis X oligosaccharides: versatile roles in cell–cell interaction. J Cell Biol. 1999;147(3):467–470. 10545492 10.1083/jcb.147.3.467 PMC2151194

[45] Shi H , Zhang J , Han X , Li H , Xie M , Sun Y , et al. Recruited monocytic myeloid‐derived suppressor cells promote the arrest of tumor cells in the premetastatic niche through an IL‐1β‐mediated increase in E‐selectin expression. Int J Cancer. 2017;140(6):1370–1383. 27885671 10.1002/ijc.30538

[46] Labelle M , Begum S , Hynes RO . Platelets guide the formation of early metastatic niches. Proc Natl Acad Sci USA. 2014;111(30):E3053–E3061. 25024172 10.1073/pnas.1411082111 PMC4121772

[47] Walker C , Mojares E , del Río Hernández A . Role of extracellular matrix in development and cancer progression. Int J Mol Sci. 2018;19(10):3028. 30287763 10.3390/ijms19103028 PMC6213383

[48] Binnemars‐Postma K , Bansal R , Storm G , Prakash J . Targeting the Stat6 pathway in tumor‐associated macrophages reduces tumor growth and metastatic niche formation in breast cancer. FASEB J. 2018;32(2):969–978. 29066614 10.1096/fj.201700629R

[49] Ludwig S , Floros T , Theodoraki MN , Hong CS , Jackson EK , Lang S , et al. Suppression of lymphocyte functions by plasma exosomes correlates with disease activity in patients with head and neck cancer. Clin Cancer Res. 2017;23(16):4843–4854. 28400428 10.1158/1078-0432.CCR-16-2819 PMC5559308

[50] Hoshino A , Costa‐Silva B , Shen TL , Rodrigues G , Hashimoto A , Tesic Mark M , et al. Tumour exosome integrins determine organotropic metastasis. Nature. 2015;527(7578):329–335. 26524530 10.1038/nature15756 PMC4788391

[51] Aguirre‐Ghiso JA . Models, mechanisms and clinical evidence for cancer dormancy. Nat Rev Cancer. 2007;7(11):834–846. 17957189 10.1038/nrc2256 PMC2519109

[52] Ghiso JAA , Kovalski K , Ossowski L . Tumor dormancy induced by downregulation of urokinase receptor in human carcinoma involves integrin and MAPK signaling. J Cell Biol. 1999;147(1):89–104. 10508858 10.1083/jcb.147.1.89 PMC2164973

[53] Indraccolo S , Stievano L , Minuzzo S , Tosello V , Esposito G , Piovan E , et al. Interruption of tumor dormancy by a transient angiogenic burst within the tumor microenvironment. Proc Natl Acad Sci USA. 2006;103(11):4216–4221. 16537511 10.1073/pnas.0506200103 PMC1449673

[54] Albrengues J , Shields MA , Ng D , Park CG , Ambrico A , Poindexter ME , et al. Neutrophil extracellular traps produced during inflammation awaken dormant cancer cells in mice. Science. 2018;361(6409):eaao4227. 30262472 10.1126/science.aao4227 PMC6777850

[55] Schlesinger M . Role of platelets and platelet receptors in cancer metastasis. J Hematol Oncol. 2018;11(1):1–15. 30305116 10.1186/s13045-018-0669-2 PMC6180572

[56] Labelle M , Begum S , Hynes RO . Direct signaling between platelets and cancer cells induces an epithelial‐mesenchymal‐like transition and promotes metastasis. Cancer Cell. 2011;20(5):576–590. 22094253 10.1016/j.ccr.2011.09.009 PMC3487108

[57] Egan K , Cooke N , Kenny D . Living in shear: platelets protect cancer cells from shear induced damage. Clin Exp Metastasis. 2014;31:697–704. 24942131 10.1007/s10585-014-9660-7

[58] Nieswandt B , Hafner M , Echtenacher B , Männel DN . Lysis of tumor cells by natural killer cells in mice is impeded by platelets. Cancer Res. 1999;59(6):1295–1300. 10096562

[59] Schumacher D , Strilic B , Sivaraj KK , Wettschureck N , Offermanns S . Platelet‐derived nucleotides promote tumor‐cell transendothelial migration and metastasis via P2Y2 receptor. Cancer Cell. 2013;24(1):130–137. 23810565 10.1016/j.ccr.2013.05.008

[60] Szczerba BM , Castro‐Giner F , Vetter M , Krol I , Gkountela S , Landin J , et al. Neutrophils escort circulating tumour cells to enable cell cycle progression. Nature. 2019;566(7745):553–557. 30728496 10.1038/s41586-019-0915-y

[61] Masucci MT , Minopoli M , del Vecchio S , Carriero MV . The emerging role of neutrophil extracellular traps (NETs) in tumor progression and metastasis. Front Immunol. 2020;11:1749. 33042107 10.3389/fimmu.2020.01749 PMC7524869

[62] Najmeh S , Cools‐Lartigue J , Rayes RF , Gowing S , Vourtzoumis P , Bourdeau F , et al. Neutrophil extracellular traps sequester circulating tumor cells via β1‐integrin mediated interactions. Int J Cancer. 2017;140(10):2321–2330. 28177522 10.1002/ijc.30635

[63] Spiegel A , Brooks MW , Houshyar S , Reinhardt F , Ardolino M , Fessler E , et al. Neutrophils suppress intraluminal NK cell–mediated tumor cell clearance and enhance extravasation of disseminated carcinoma cells. Cancer Discov. 2016;6(6):630–649. 27072748 10.1158/2159-8290.CD-15-1157 PMC4918202

[64] Hamilton G , Rath B , Klameth L , Hochmair MJ . Small cell lung cancer: recruitment of macrophages by circulating tumor cells. Onco Targets Ther. 2016;5(3):e1093277. 10.1080/2162402X.2015.1093277 PMC4839345 27141354

[65] Osmulski PA , Cunsolo A , Chen M , Qian Y , Lin CL , Hung CN , et al. Contacts with macrophages promote an aggressive nanomechanical phenotype of circulating tumor cells in prostate cancer. Cancer Res. 2021;81(15):4110–4123. 34045187 10.1158/0008-5472.CAN-20-3595 PMC8367292

[66] Shabo I , Midtbö K , Andersson H , Åkerlund E , Olsson H , Wegman P , et al. Macrophage traits in cancer cells are induced by macrophage‐cancer cell fusion and cannot be explained by cellular interaction. BMC Cancer. 2015;15(1):1–11. 26585897 10.1186/s12885-015-1935-0 PMC4653907

[67] Gast CE , Silk AD , Zarour L , Riegler L , Burkhart JG , Gustafson KT , et al. Cell fusion potentiates tumor heterogeneity and reveals circulating hybrid cells that correlate with stage and survival. Sci Adv. 2018;4(9):eaat7828. 30214939 10.1126/sciadv.aat7828 PMC6135550

[68] Clawson GA , Matters GL , Xin P , McGovern C , Wafula E , dePamphilis C , et al. “Stealth dissemination” of macrophage‐tumor cell fusions cultured from blood of patients with pancreatic ductal adenocarcinoma. PLoS One. 2017;12(9):e0184451. 28957348 10.1371/journal.pone.0184451 PMC5619717

[69] Liu Q , Liao Q , Zhao Y . Myeloid‐derived suppressor cells (MDSC) facilitate distant metastasis of malignancies by shielding circulating tumor cells (CTC) from immune surveillance. Med Hypotheses. 2016;87:34–39. 26826638 10.1016/j.mehy.2015.12.007

[70] Sprouse ML , Welte T , Boral D , Liu HN , Yin W , Vishnoi M , et al. PMN‐MDSCs enhance CTC metastatic properties through reciprocal interactions via ROS/notch/nodal signaling. Int J Mol Sci. 2019;20(8):1916. 31003475 10.3390/ijms20081916 PMC6514876

[71] Rashida Gnanaprakasam JN , Wu R , Wang R . Metabolic reprogramming in modulating T cell reactive oxygen species generation and antioxidant capacity. Front Immunol. 2018;9:1075. 29868027 10.3389/fimmu.2018.01075 PMC5964129

[72] Alfaro C , Teijeira A , Oñate C , Pérez G , Sanmamed MF , Andueza MP , et al. Tumor‐produced interleukin‐8 attracts human myeloid‐derived suppressor cells and elicits extrusion of neutrophil extracellular traps (NETs). Clin Cancer Res. 2016;22(15):3924–3936. 26957562 10.1158/1078-0432.CCR-15-2463

[73] Duda DG , Duyverman AMMJ , Kohno M , Snuderl M , Steller EJA , Fukumura D , et al. Malignant cells facilitate lung metastasis by bringing their own soil. Proc Natl Acad Sci. 2010;107(50):21677–21682. 21098274 10.1073/pnas.1016234107 PMC3003109

[74] Ortiz‐Otero N , Clinch AB , Hope J , Wang W , Reinhart‐King CA , King MR . Cancer associated fibroblasts confer shear resistance to circulating tumor cells during prostate cancer metastatic progression. Oncotarget. 2020;11(12):1037–1050. 32256977 10.18632/oncotarget.27510 PMC7105166

[75] Hurtado P , Martínez‐Pena I , Piñeiro R . Dangerous liaisons: circulating tumor cells (CTCs) and cancer‐associated fibroblasts (CAFs). Cancer. 2020;12(10):2861. 10.3390/cancers12102861 PMC7599894 33027902

[76] Heeke S , Mograbi B , Alix‐Panabières C , Hofman P . Never travel alone: the crosstalk of circulating tumor cells and the blood microenvironment. Cells. 2019;8(7):714. 31337010 10.3390/cells8070714 PMC6678604

[77] Watson NF , Ramage JM , Madjd Z , Spendlove I , Ellis IO , Scholefield JH , et al. Immunosurveillance is active in colorectal cancer as downregulation but not complete loss of MHC class I expression correlates with a poor prognosis. Int J Cancer. 2006;118(1):6–10. 16003753 10.1002/ijc.21303

[78] Aptsiauri N , Cabrera T , Mendez R , Garcia‐Lora A , Ruiz‐Cabello F , Garrido F . Role of altered expression of HLA class I molecules in cancer progression. Immune‐Mediated Diseases: From Theory to Therapy; 2007. p. 123–131. 10.1007/978-0-387-72005-0_13 17712999

[79] Pantel K , Schlimok G , Kutter D , Schaller G , Genz T , Wiebecke B , et al. Frequent down‐regulation of major histocompatibility class I antigen expression on individual micrometastatic carcinoma cells. Cancer Res. 1991;51(17):4712–4715. 1873815

[80] Waldhauer I , Steinle A . NK cells and cancer immunosurveillance. Oncogene. 2008;27(45):5932–5943. 18836474 10.1038/onc.2008.267

[81] Placke T , Örgel M , Schaller M , Jung G , Rammensee HG , Kopp HG , et al. Platelet‐derived MHC class I confers a pseudonormal phenotype to cancer cells that subverts the antitumor reactivity of natural killer immune cells. Cancer Res. 2012;72(2):440–448. 22127925 10.1158/0008-5472.CAN-11-1872

[82] Wu M‐S , Li CH , Ruppert JG , Chang CC . Cytokeratin 8‐MHC class I interactions: a potential novel immune escape phenotype by a lymph node metastatic carcinoma cell line. Biochem Biophys Res Commun. 2013;441(3):618–623. 24183726 10.1016/j.bbrc.2013.10.105

[83] Cluxton CD , Spillane C , O'Toole SA , Sheils O , Gardiner CM , O'Leary JJ . Suppression of natural killer cell NKG2D and CD226 anti‐tumour cascades by platelet cloaked cancer cells: implications for the metastatic cascade. PLoS One. 2019;14(3):e0211538. 30908480 10.1371/journal.pone.0211538 PMC6433214

[84] Oldenborg P‐A , Zheleznyak A , Fang YF , Lagenaur CF , Gresham HD , Lindberg FP . Role of CD47 as a marker of self on red blood cells. Science. 2000;288(5473):2051–2054. 10856220 10.1126/science.288.5473.2051

[85] Steinert G , Schölch S , Niemietz T , Iwata N , García SA , Behrens B , et al. Immune escape and survival mechanisms in circulating tumor cells of colorectal cancer. Cancer Res. 2014;74(6):1694–1704. 24599131 10.1158/0008-5472.CAN-13-1885

[86] Torres JA , Brito ABC , Silva VS , Messias IM , Braun AC , Ruano APC , et al. CD47 expression in circulating tumor cells and circulating tumor microemboli from non‐small cell lung cancer patients is a poor prognosis factor. Int J Mol Sci. 2023;24(15):11958. 37569332 10.3390/ijms241511958 PMC10419161

[87] Chikamatsu K , Tada H , Takahashi H , Kuwabara‐Yokobori Y , Ishii H , Ida S , et al. Expression of immune‐regulatory molecules in circulating tumor cells derived from patients with head and neck squamous cell carcinoma. Oral Oncol. 2019;89:34–39. 30732956 10.1016/j.oraloncology.2018.12.002

[88] Papadaki MA , Koutsopoulos AV , Tsoulfas PG , Lagoudaki E , Aggouraki D , Monastirioti A , et al. Clinical relevance of immune checkpoints on circulating tumor cells in breast cancer. Cancer. 2020;12(2):376. 10.3390/cancers12020376 PMC7072621 32041353

[89] Gruber I , Landenberger N , Staebler A , Hahn M , Wallwiener D , Fehm T . Relationship between circulating tumor cells and peripheral T‐cells in patients with primary breast cancer. Anticancer Res. 2013;33(5):2233–2238. 23645781

[90] Owen‐Schaub L , Chan H , Cusack JC , Roth J , Hill LL . Fas and Fas ligand interactions in malignant disease. Int J Oncol. 2000;17(1):5–17. 10853011

[91] Hallermalm K , de Geer A , Kiessling R , Levitsky V , Levitskaya J . Autocrine secretion of Fas ligand shields tumor cells from Fas‐mediated killing by cytotoxic lymphocytes. Cancer Res. 2004;64(18):6775–6782. 15374996 10.1158/0008-5472.CAN-04-0508

[92] Keir ME , Butte MJ , Freeman GJ , Sharpe AH . PD‐1 and its ligands in tolerance and immunity. Annu Rev Immunol. 2008;26:677–704. 18173375 10.1146/annurev.immunol.26.021607.090331 PMC10637733

[93] Strati A , Koutsodontis G , Papaxoinis G , Angelidis I , Zavridou M , Economopoulou P , et al. Prognostic significance of PD‐L1 expression on circulating tumor cells in patients with head and neck squamous cell carcinoma. Ann Oncol. 2017;28(8):1923–1933. 28838214 10.1093/annonc/mdx206

[94] Nicolazzo C , Raimondi C , Mancini ML , Caponnetto S , Gradilone A , Gandini O , et al. Monitoring PD‐L1 positive circulating tumor cells in non‐small cell lung cancer patients treated with the PD‐1 inhibitor nivolumab. Sci Rep. 2016;6(1):31726. 27553175 10.1038/srep31726 PMC4995431

[95] Mazel M , Jacot W , Pantel K , Bartkowiak K , Topart D , Cayrefourcq L , et al. Frequent expression of PD‐L1 on circulating breast cancer cells. Mol Oncol. 2015;9(9):1773–1782. 26093818 10.1016/j.molonc.2015.05.009 PMC5528721

[96] Yang Y . Cancer immunotherapy: harnessing the immune system to battle cancer. J Clin Invest. 2015;125(9):3335–3337. 26325031 10.1172/JCI83871 PMC4588312

[97] Wainberg ZA , Fuchs CS , Tabernero J , Shitara K , Muro K , van Cutsem E , et al. Efficacy of pembrolizumab monotherapy for advanced gastric/gastroesophageal junction cancer with programmed death ligand 1 combined positive score≥ 10. Clin Cancer Res. 2021;27(7):1923–1931. 33446564 10.1158/1078-0432.CCR-20-2980

[98] Pedersen KS , Foster NR , Overman MJ , Boland PM , Kim SS , Arrambide KA , et al. ZEBRA: a multicenter phase II study of pembrolizumab in patients with advanced small‐bowel adenocarcinoma. Clin Cancer Res. 2021;27(13):3641–3648. 33883178 10.1158/1078-0432.CCR-21-0159

[99] Robert C , Schachter J , Long GV , Arance A , Grob JJ , Mortier L , et al. Pembrolizumab versus ipilimumab in advanced melanoma. N Engl J Med. 2015;372(26):2521–2532. 25891173 10.1056/NEJMoa1503093

[100] Schellens JH et al. Pembrolizumab for previously treated advanced cervical squamous cell cancer: preliminary results from the phase 2 KEYNOTE‐158 study. Vol 385. Am Soc Clin Oncol; 2017. p. 1856–1867.

[101] McDermott DF , Lee JL , Ziobro M , Suarez C , Langiewicz P , Matveev VB , et al. Open‐label, single‐arm, phase II study of pembrolizumab monotherapy as first‐line therapy in patients with advanced non–clear cell renal cell carcinoma. J Clin Oncol. 2021;39(9):1029–1039. 33529058 10.1200/JCO.20.02365 PMC8078262

[102] Gettinger S , Rizvi NA , Chow LQ , Borghaei H , Brahmer J , Ready N , et al. Nivolumab monotherapy for first‐line treatment of advanced non–small‐cell lung cancer. J Clin Oncol. 2016;34(25):2980–2987. 27354485 10.1200/JCO.2016.66.9929 PMC5569692

[103] Sharma P , Retz M , Siefker‐Radtke A , Baron A , Necchi A , Bedke J , et al. Nivolumab in metastatic urothelial carcinoma after platinum therapy (CheckMate 275): a multicentre, single‐arm, phase 2 trial. Lancet Oncol. 2017;18(3):312–322. 28131785 10.1016/S1470-2045(17)30065-7

[104] El‐Khoueiry AB , Sangro B , Yau T , Crocenzi TS , Kudo M , Hsu C , et al. Nivolumab in patients with advanced hepatocellular carcinoma (CheckMate 040): an open‐label, non‐comparative, phase 1/2 dose escalation and expansion trial. Lancet. 2017;389(10088):2492–2502. 28434648 10.1016/S0140-6736(17)31046-2 PMC7539326

[105] Wolchok JD , Hodi FS , Weber JS , Allison JP , Urba WJ , Robert C , et al. Development of ipilimumab: a novel immunotherapeutic approach for the treatment of advanced melanoma. Ann N Y Acad Sci. 2013;1291(1):1–13. 23772560 10.1111/nyas.12180 PMC3910157

[106] Koller KM , Mackley HB , Liu J , Wagner H , Talamo G , Schell TD , et al. Improved survival and complete response rates in patients with advanced melanoma treated with concurrent ipilimumab and radiotherapy versus ipilimumab alone. Cancer Biol Ther. 2017;18(1):36–42. 27905824 10.1080/15384047.2016.1264543 PMC5323007

[107] Zaretsky JM , Garcia‐Diaz A , Shin DS , Escuin‐Ordinas H , Hugo W , Hu‐Lieskovan S , et al. Mutations associated with acquired resistance to PD‐1 blockade in melanoma. N Engl J Med. 2016;375(9):819–829. 27433843 10.1056/NEJMoa1604958 PMC5007206

[108] Chowell D , Morris LGT , Grigg CM , Weber JK , Samstein RM , Makarov V , et al. Patient HLA class I genotype influences cancer response to checkpoint blockade immunotherapy. Science. 2018;359(6375):582–587. 29217585 10.1126/science.aao4572 PMC6057471

[109] Karlsson AK , Saleh SN . Checkpoint inhibitors for malignant melanoma: a systematic review and meta‐analysis. Clin Cosmet Investig Dermatol. 2017;10:325–339. 10.2147/CCID.S120877 PMC5580705 28883738

[110] Gandhi L , Rodríguez‐Abreu D , Gadgeel S , Esteban E , Felip E , de Angelis F , et al. Pembrolizumab plus chemotherapy in metastatic non–small‐cell lung cancer. N Engl J Med. 2018;378(22):2078–2092. 29658856 10.1056/NEJMoa1801005

[111] Colombo N , Dubot C , Lorusso D , Caceres MV , Hasegawa K , Shapira‐Frommer R , et al. Pembrolizumab for persistent, recurrent, or metastatic cervical cancer. N Engl J Med. 2021;385(20):1856–1867. 34534429 10.1056/NEJMoa2112435

[112] Eskander RN , Sill MW , Beffa L , Moore RG , Hope JM , Musa FB , et al. Pembrolizumab plus chemotherapy in advanced endometrial cancer. N Engl J Med. 2023;388:2159–2170. 36972022 10.1056/NEJMoa2302312 PMC10351614

[113] Cabrera T , Lara E , Romero JM , Maleno I , Real LM , Ruiz‐Cabello F , et al. HLA class I expression in metastatic melanoma correlates with tumor development during autologous vaccination. Cancer Immunol Immunother. 2007;56:709–717. 16960691 10.1007/s00262-006-0226-7 PMC11030676

[114] Mariathasan S , Turley SJ , Nickles D , Castiglioni A , Yuen K , Wang Y , et al. TGFβ attenuates tumour response to PD‐L1 blockade by contributing to exclusion of T cells. Nature. 2018;554(7693):544–548. 29443960 10.1038/nature25501 PMC6028240

[115] Zer A , Sung MR , Walia P , Khoja L , Maganti M , Labbe C , et al. Correlation of neutrophil to lymphocyte ratio and absolute neutrophil count with outcomes with PD‐1 axis inhibitors in patients with advanced non–small‐cell lung cancer. Clin Lung Cancer. 2018;19(5):426–434.e1. 29803574 10.1016/j.cllc.2018.04.008

[116] Rosenberg SA , Yang JC , Restifo NP . Cancer immunotherapy: moving beyond current vaccines. Nat Med. 2004;10(9):909–915. 15340416 10.1038/nm1100 PMC1435696

[117] Nagorsen D , Thiel E . Clinical and immunologic responses to active specific cancer vaccines in human colorectal cancer. Clin Cancer Res. 2006;12(10):3064–3069. 16707603 10.1158/1078-0432.CCR-05-2788

[118] Lopez JS , Camidge R , Iafolla M , Rottey S , Schuler M , Hellmann M , et al. A phase Ib study to evaluate RO7198457, an individualized neoantigen specific immunoTherapy (iNeST), in combination with atezolizumab in patients with locally advanced or metastatic solid tumors. Cancer Res. 2020;80(16 Suppl):CT301.

[119] Burris HA , Patel MR , Cho DC , Clarke JM , Gutierrez M , Zaks TZ , et al. A phase I multicenter study to assess the safety, tolerability, and immunogenicity of mRNA‐4157 alone in patients with resected solid tumors and in combination with pembrolizumab in patients with unresectable solid tumors. Am Soc Clin Oncol. 2019;37:2523.

[120] Smith FO , Downey SG , Klapper JA , Yang JC , Sherry RM , Royal RE , et al. Treatment of metastatic melanoma using interleukin‐2 alone or in conjunction with vaccines. Clin Cancer Res. 2008;14(17):5610–5618. 18765555 10.1158/1078-0432.CCR-08-0116 PMC2656367

[121] Schwartzentruber DJ , Lawson DH , Richards JM , Conry RM , Miller DM , Treisman J , et al. gp100 peptide vaccine and interleukin‐2 in patients with advanced melanoma. N Engl J Med. 2011;364(22):2119–2127. 21631324 10.1056/NEJMoa1012863 PMC3517182

[122] Turtle CJ , Hanafi LA , Berger C , Hudecek M , Pender B , Robinson E , et al. Immunotherapy of non‐Hodgkin's lymphoma with a defined ratio of CD8^+^ and CD4^+^ CD19‐specific chimeric antigen receptor–modified T cells. Sci Transl Med. 2016;8(355):355ra116. 10.1126/scitranslmed.aaf8621 PMC5045301 27605551

[123] Brentjens RJ , Davila ML , Riviere I , Park J , Wang X , Cowell LG , et al. CD19‐targeted T cells rapidly induce molecular remissions in adults with chemotherapy‐refractory acute lymphoblastic leukemia. Sci Transl Med. 2013;5(177):177ra38. 10.1126/scitranslmed.3005930 PMC3742551 23515080

[124] Maude SL , Frey N , Shaw PA , Aplenc R , Barrett DM , Bunin NJ , et al. Chimeric antigen receptor T cells for sustained remissions in leukemia. N Engl J Med. 2014;371(16):1507–1517. 25317870 10.1056/NEJMoa1407222 PMC4267531

[125] Hou B , Tang Y , Li W , Zeng Q , Chang D . Efficiency of CAR‐T therapy for treatment of solid tumor in clinical trials: a meta‐analysis. Dis Markers. 2019;2019:1–11. 10.1155/2019/3425291 PMC6388318 30886654

[126] Ahmed N , Brawley VS , Hegde M , Robertson C , Ghazi A , Gerken C , et al. Human epidermal growth factor receptor 2 (HER2)–specific chimeric antigen receptor–modified T cells for the immunotherapy of HER2‐positive sarcoma. J Clin Oncol. 2015;33(15):1688–1696. 25800760 10.1200/JCO.2014.58.0225 PMC4429176

[127] Sotillo E , Barrett DM , Black KL , Bagashev A , Oldridge D , Wu G , et al. Convergence of acquired mutations and alternative splicing of CD19 enables resistance to CART‐19 immunotherapy. Cancer Discov. 2015;5(12):1282–1295. 26516065 10.1158/2159-8290.CD-15-1020 PMC4670800

[128] Hegde M , Mukherjee M , Grada Z , Pignata A , Landi D , Navai SA , et al. Tandem CAR T cells targeting HER2 and IL13Rα2 mitigate tumor antigen escape. J Clin Invest. 2016;126(8):3036–3052. 27427982 10.1172/JCI83416 PMC4966331

[129] Neelapu SS , Tummala S , Kebriaei P , Wierda W , Gutierrez C , Locke FL , et al. Chimeric antigen receptor T‐cell therapy—assessment and management of toxicities. Nat Rev Clin Oncol. 2018;15(1):47–62. 28925994 10.1038/nrclinonc.2017.148 PMC6733403

[130] Esfandiari A , Cassidy S , Webster RM . Bispecific antibodies in oncology. Nat Rev Drug Discov. 2022;21(6):411–412. 35246638 10.1038/d41573-022-00040-2

[131] Dhillon S . Tebentafusp: first approval. Drugs. 2022;82(6):703–710. 35364798 10.1007/s40265-022-01704-4

[132] Nathan P , Hassel JC , Rutkowski P , Baurain JF , Butler MO , Schlaak M , et al. Overall survival benefit with tebentafusp in metastatic uveal melanoma. N Engl J Med. 2021;385(13):1196–1206. 34551229 10.1056/NEJMoa2103485

[133] Moreau P , Garfall AL , van de Donk NWCJ , Nahi H , San‐Miguel JF , Oriol A , et al. Teclistamab in relapsed or refractory multiple myeloma. N Engl J Med. 2022;387(6):495–505. 35661166 10.1056/NEJMoa2203478 PMC10587778

[134] Sinkala E , Sollier‐Christen E , Renier C , Rosàs‐Canyelles E , Che J , Heirich K , et al. Profiling protein expression in circulating tumour cells using microfluidic western blotting. Nat Commun. 2017;8(1):14622. 28332571 10.1038/ncomms14622 PMC5376644

[135] Zhu Y , Podolak J , Zhao R , Shukla AK , Moore RJ , Thomas GV , et al. Proteome profiling of 1 to 5 spiked circulating tumor cells isolated from whole blood using immunodensity enrichment, laser capture microdissection, nanodroplet sample processing, and ultrasensitive nanoLC–MS. Anal Chem. 2018;90(20):11756–11759. 30269481 10.1021/acs.analchem.8b03268 PMC6686195

[136] Payne K , Brooks J , Batis N , Khan N , el‐Asrag M , Nankivell P , et al. Feasibility of mass cytometry proteomic characterisation of circulating tumour cells in head and neck squamous cell carcinoma for deep phenotyping. Br J Cancer. 2023;129(10):1590–1598. 37735243 10.1038/s41416-023-02428-2 PMC10645808

[137] Donato C , Buczak K , Schmidt A , Aceto N . Mass spectrometry analysis of circulating breast cancer cells from a xenograft mouse model. STAR Protoc. 2021;2(2):100480. 33982014 10.1016/j.xpro.2021.100480 PMC8082161

[138] Eslami‐S Z , Cortés‐Hernández LE , Thomas F , Pantel K , Alix‐Panabières C . Functional analysis of circulating tumour cells: the KEY to understand the biology of the metastatic cascade. Br J Cancer. 2022;127(5):800–810. 35484215 10.1038/s41416-022-01819-1 PMC9427839

[139] Stoecklein NH , Fischer JC , Niederacher D , Terstappen LWMM . Challenges for CTC‐based liquid biopsies: low CTC frequency and diagnostic leukapheresis as a potential solution. Expert Rev Mol Diagn. 2016;16(2):147–164. 26587751 10.1586/14737159.2016.1123095

[140] Klameth L , Rath B , Hochmaier M , Moser D , Redl M , Mungenast F , et al. Small cell lung cancer: model of circulating tumor cell tumorospheres in chemoresistance. Sci Rep. 2017;7(1):5337. 28706293 10.1038/s41598-017-05562-z PMC5509650

[141] Cayrefourcq L , Mazard T , Joosse S , Solassol J , Ramos J , Assenat E , et al. Establishment and characterization of a cell line from human circulating colon cancer cells. Cancer Res. 2015;75(5):892–901. 25592149 10.1158/0008-5472.CAN-14-2613

[142] Shapiro IE , Bassani‐Sternberg M . The impact of immunopeptidomics: from basic research to clinical implementation. Semin Immunol. 2023;66:101727. 36764021 10.1016/j.smim.2023.101727

[143] Pishesha N , Harmand TJ , Ploegh HL . A guide to antigen processing and presentation. Nat Rev Immunol. 2022;22(12):751–764. 35418563 10.1038/s41577-022-00707-2

[144] Neefjes J , Jongsma MLM , Paul P , Bakke O . Towards a systems understanding of MHC class I and MHC class II antigen presentation. Nat Rev Immunol. 2011;11(12):823–836. 22076556 10.1038/nri3084

[145] Roche PA , Furuta K . The ins and outs of MHC class II‐mediated antigen processing and presentation. Nat Rev Immunol. 2015;15(4):203–216. 25720354 10.1038/nri3818 PMC6314495

[146] Meyer VS , Drews O , Günder M , Hennenlotter J , Rammensee HG , Stevanovic S . Identification of natural MHC class II presented phosphopeptides and tumor‐derived MHC class I phospholigands. J Proteome Res. 2009;8(7):3666–3674. 19415920 10.1021/pr800937k

[147] Zarling AL , Polefrone JM , Evans AM , Mikesh LM , Shabanowitz J , Lewis ST , et al. Identification of class I MHC‐associated phosphopeptides as targets for cancer immunotherapy. Proc Natl Acad Sci USA. 2006;103(40):14889–14894. 17001009 10.1073/pnas.0604045103 PMC1595446

[148] Cobbold M , De La Peña H , Norris A , Polefrone JM , Qian J , English AM , et al. MHC class I–associated phosphopeptides are the targets of memory‐like immunity in leukemia. Sci Transl Med. 2013;5(203):203ra125. 10.1126/scitranslmed.3006061 PMC4071620 24048523

[149] Depontieu FR , Qian J , Zarling AL , McMiller TL , Salay TM , Norris A , et al. Identification of tumor‐associated, MHC class II‐restricted phosphopeptides as targets for immunotherapy. Proc Natl Acad Sci USA. 2009;106(29):12073–12078. 19581576 10.1073/pnas.0903852106 PMC2715484

[150] Wang Y , Chen R , Gan Y , Ying S . The roles of PAD2‐and PAD4‐mediated protein citrullination catalysis in cancers. Int J Cancer. 2021;148(2):267–276. 33459350 10.1002/ijc.33205

[151] Katayama H , Kobayashi M , Irajizad E , Sevillano AM , Patel N , Mao X , et al. Protein citrullination as a source of cancer neoantigens. J Immunother Cancer. 2021;9(6):e002549. 34112737 10.1136/jitc-2021-002549 PMC8194337

[152] Symonds P , Marcu A , Cook KW , Metheringham RL , Durrant LG , Brentville VA . Citrullinated epitopes identified on tumour MHC class II by peptide elution stimulate both regulatory and Th1 responses and require careful selection for optimal anti‐tumour responses. Front Immunol. 2021;12:764462. 34858415 10.3389/fimmu.2021.764462 PMC8630742

[153] Brentville VA , Symonds P , Cook KW , Daniels I , Pitt T , Gijon M , et al. T cell repertoire to citrullinated self‐peptides in healthy humans is not confined to the HLA‐DR SE alleles; targeting of citrullinated self‐peptides presented by HLA‐DP4 for tumour therapy. Onco Targets Ther. 2019;8(5):e1576490. 10.1080/2162402X.2019.1576490 PMC6492960 31069134

[154] Brentville V , Vankemmelbeke M , Metheringham RL , Durrant LG . Post‐translational modifications such as citrullination are excellent targets for cancer therapy. Semin Immunol. 2020;47:101393. 31932199 10.1016/j.smim.2020.101393

[155] Haen SP , Löffler MW , Rammensee HG , Brossart P . Towards new horizons: characterization, classification and implications of the tumour antigenic repertoire. Nat Rev Clin Oncol. 2020;17(10):595–610. 32572208 10.1038/s41571-020-0387-x PMC7306938

[156] Türeci Ö , Vormehr M , Diken M , Kreiter S , Huber C , Sahin U . Targeting the heterogeneity of cancer with individualized neoepitope vaccines. Clin Cancer Res. 2016;22(8):1885–1896. 27084742 10.1158/1078-0432.CCR-15-1509

[157] Duan F , Duitama J , al Seesi S , Ayres CM , Corcelli SA , Pawashe AP , et al. Genomic and bioinformatic profiling of mutational neoepitopes reveals new rules to predict anticancer immunogenicity. J Exp Med. 2014;211(11):2231–2248. 25245761 10.1084/jem.20141308 PMC4203949

[158] Shao XM , Bhattacharya R , Huang J , Sivakumar IKA , Tokheim C , Zheng L , et al. High‐throughput prediction of MHC class I and II neoantigens with MHCnuggets. Cancer Immunol Res. 2020;8(3):396–408. 31871119 10.1158/2326-6066.CIR-19-0464 PMC7056596

[159] Jurtz V , Paul S , Andreatta M , Marcatili P , Peters B , Nielsen M . NetMHCpan‐4.0: improved peptide–MHC class I interaction predictions integrating eluted ligand and peptide binding affinity data. J Immunol. 2017;199(9):3360–3368. 28978689 10.4049/jimmunol.1700893 PMC5679736

[160] Racle J , Michaux J , Rockinger GA , Arnaud M , Bobisse S , Chong C , et al. Robust prediction of HLA class II epitopes by deep motif deconvolution of immunopeptidomes. Nat Biotechnol. 2019;37(11):1283–1286. 31611696 10.1038/s41587-019-0289-6

[161] Cox J , Mann M . MaxQuant enables high peptide identification rates, individualized ppb‐range mass accuracies and proteome‐wide protein quantification. Nat Biotechnol. 2008;26(12):1367–1372. 19029910 10.1038/nbt.1511

[162] Irving M , Zoete V , Bassani‐Sternberg M , Coukos G . A roadmap for driving CAR T cells toward the oncogenic immunopeptidome. Cancer Cell. 2022;40(1):20–22. 35016027 10.1016/j.ccell.2021.12.011

[163] Chong C , Müller M , Pak HS , Harnett D , Huber F , Grun D , et al. Integrated proteogenomic deep sequencing and analytics accurately identify non‐canonical peptides in tumor immunopeptidomes. Nat Commun. 2020;11(1):1293. 32157095 10.1038/s41467-020-14968-9 PMC7064602

[164] Feola S , Chiaro J , Cerullo V . Integrating immunopeptidome analysis for the design and development of cancer vaccines. Semin Immunol. 2023;67:101750. 37003057 10.1016/j.smim.2023.101750

[165] Pak H , Michaux J , Huber F , Chong C , Stevenson BJ , Müller M , et al. Sensitive immunopeptidomics by leveraging available large‐scale multi‐HLA spectral libraries, data‐independent acquisition, and MS/MS prediction. Mol Cell Proteomics. 2021;20:100080. 33845167 10.1016/j.mcpro.2021.100080 PMC8724634

[166] Pinho SS , Reis CA . Glycosylation in cancer: mechanisms and clinical implications. Nat Rev Cancer. 2015;15(9):540–555. 26289314 10.1038/nrc3982

[167] Hakomori S‐I . Tumor malignancy defined by aberrant glycosylation and sphingo (glyco) lipid metabolism. Cancer Res. 1996;56(23):5309–5318. 8968075

[168] Magalhães A , Duarte HO , Reis CA . Aberrant glycosylation in cancer: a novel molecular mechanism controlling metastasis. Cancer Cell. 2017;31(6):733–735. 28609653 10.1016/j.ccell.2017.05.012

[169] Brown GD , Willment JA , Whitehead L . C‐type lectins in immunity and homeostasis. Nat Rev Immunol. 2018;18(6):374–389. 29581532 10.1038/s41577-018-0004-8

[170] Yang Z , Harris LE , Palmer‐Toy DE , Hancock WS . Multilectin affinity chromatography for characterization of multiple glycoprotein biomarker candidates in serum from breast cancer patients. Clin Chem. 2006;52(10):1897–1905. 16916992 10.1373/clinchem.2005.065862

[171] Hirabayashi J , Yamada M , Kuno A , Tateno H . Lectin microarrays: concept, principle and applications. Chem Soc Rev. 2013;42(10):4443–4458. 23443201 10.1039/c3cs35419a

[172] McCoy JP Jr , Varani J , Goldstein IJ . Enzyme‐linked lectin assay (ELLA): II. Detection of carbohydrate groups on the surface of unfixed cells. Exp Cell Res. 1984;151(1):96–103. 6698125 10.1016/0014-4827(84)90359-8

[173] Cui H , Lin Y , Yue L , Zhao X , Liu J . Differential expression of the α2, 3‐sialic acid residues in breast cancer is associated with metastatic potential. Oncol Rep. 2011;25(5):1365–1371. 21344161 10.3892/or.2011.1192

[174] Bassagañas S , Pérez‐Garay M , Peracaula R . Cell surface sialic acid modulates extracellular matrix adhesion and migration in pancreatic adenocarcinoma cells. Pancreas. 2014;43(1):109–117. 23921962 10.1097/MPA.0b013e31829d9090

[175] Wang P‐H , Lee WL , Juang CM , Yang YH , Lo WH , Lai CR , et al. Altered mRNA expressions of sialyltransferases in ovarian cancers. Gynecol Oncol. 2005;99(3):631–639. 16112178 10.1016/j.ygyno.2005.07.016

[176] Tanaka F , Otake Y , Nakagawa T , Kawano Y , Miyahara R , Li M , et al. Prognostic significance of polysialic acid expression in resected non‐small cell lung cancer. Cancer Res. 2001;61(4):1666–1670. 11245481

[177] Adams OJ , Stanczak MA , von Gunten S , Läubli H . Targeting sialic acid–Siglec interactions to reverse immune suppression in cancer. Glycobiology. 2018;28(9):640–647. 29309569 10.1093/glycob/cwx108

[178] Wu Y , Huang W , Xie Y , Wang C , Luo N , Chen Y , et al. Siglec‐9, a putative immune checkpoint marker for cancer progression across multiple cancer types. Front Mol Biosci. 2022;9:743515. 35372497 10.3389/fmolb.2022.743515 PMC8968865

[179] Läubli H , Pearce OMT , Schwarz F , Siddiqui SS , Deng L , Stanczak MA , et al. Engagement of myelomonocytic Siglecs by tumor‐associated ligands modulates the innate immune response to cancer. Proc Natl Acad Sci USA. 2014;111(39):14211–14216. 25225409 10.1073/pnas.1409580111 PMC4191788

[180] Perdicchio M , Cornelissen LAM , Streng‐Ouwehand I , Engels S , Verstege MI , Boon L , et al. Tumor sialylation impedes T cell mediated anti‐tumor responses while promoting tumor associated‐regulatory T cells. Oncotarget. 2016;7(8):8771–8782. 26741508 10.18632/oncotarget.6822 PMC4891003

[181] Jandus C , Boligan KF , Chijioke O , Liu H , Dahlhaus M , Démoulins T , et al. Interactions between Siglec‐7/9 receptors and ligands influence NK cell–dependent tumor immunosurveillance. J Clin Invest. 2014;124(4):1810–1820. 24569453 10.1172/JCI65899 PMC3973073

[182] Abouleila Y , Onidani K , Ali A , Shoji H , Kawai T , Lim CT , et al. Live single cell mass spectrometry reveals cancer‐specific metabolic profiles of circulating tumor cells. Cancer Sci. 2019;110(2):697–706. 30549153 10.1111/cas.13915 PMC6361580

